# Calenduloside E Analogues Protecting H9c2 Cardiomyocytes Against H_2_O_2_-Induced Apoptosis: Design, Synthesis and Biological Evaluation

**DOI:** 10.3389/fphar.2017.00862

**Published:** 2017-11-23

**Authors:** Yu Tian, Yu-Yang Du, Hai Shang, Min Wang, Zhong-Hao Sun, Bao-Qi Wang, Di Deng, Shan Wang, Xu-Dong Xu, Gui-Bo Sun, Xiao-Bo Sun

**Affiliations:** ^1^Beijing Key Laboratory of Innovative Drug Discovery of Traditional Chinese Medicine (Natural Medicine) and Translational Medicine, Chinese Academy of Medical Sciences and Peking Union Medical College, Beijing, China; ^2^Key Laboratory of Bioactive Substances and Resources Utilization of Chinese Herbal Medicine, Ministry of Education, Chinese Academy of Medical Sciences and Peking Union Medical College, Beijing, China; ^3^Key Laboratory of Efficacy Evaluation of Chinese Medicine against Glycolipid Metabolic Disorders, State Administration of Traditional Chinese Medicine, Chinese Academy of Medical Sciences and Peking Union Medical College, Beijing, China; ^4^Zhong Guan Cun Open Laboratory of the Research and Development of Natural Medicine and Health Products, Chinese Academy of Medical Sciences and Peking Union Medical College, Beijing, China; ^5^Institute of Medicinal Plant Development, Chinese Academy of Medical Sciences and Peking Union Medical College, Beijing, China; ^6^Center of Research and Development on Life Sciences and Environment Sciences, Harbin University of Commerce, Harbin, China

**Keywords:** Calenduloside E (CE) analogues, triterpenoid saponin, cardiomyocytes, apoptosis, ROS, anti-apoptotic mechanism

## Abstract

Modulation of apoptosis is therapeutically effective in cardiomyocytes damage. Calenduloside E (CE), a naturally occurring triterpenoid saponin, is a potent anti-apoptotic agent. However, little is known about its synthetic analogues on the protective effects in apoptosis of cardiomyocytes. The present research was performed to investigate the potential protective effect of CE analogues against H_2_O_2_-induced apoptosis in H9c2 cardiomyocytes and the underlying mechanisms. Sixteen novel CE anologues have been designed, synthesized and biological evaluation. Among the 16 CE anologues, as well as the positive control CE tested, compound **5d** was the most effective in improving cardiomyocytes viability. Pretreatment with anologue **5d** inhibited ROS generation, maintained the mitochondrial membrane potential and reduced apoptotic cardiomyocytes. Moreover, exposure to H_2_O_2_ significantly increased the levels of Bax, cleaved caspase-3, and cleaved PARP, and decreased the level of Bcl-2, resulting in cell apoptosis. Pretreatment with anologue **5d** (0.02–0.5 μg/mL) dose-dependently upregulated antiapoptotic proteins and downregulated proapoptotic proteins mentioned above during H_2_O_2_-induced apoptosis. These results suggested that CE analogues provide protection to H9c2 cardiomyocytes against H_2_O_2_-induced oxidative stress and apoptosis, most likely via anti-apoptotic mechanism, and provided the basis for the further optimization of the CE analogues.

## Introduction

Cardiovascular disease is the leading cause of death worldwide for its high morbidity and mortality. According to the World Health Organization, cardiovascular diseases mortality may be expected to reach about 25 million by 2030 (Chaudhari et al., 2014; Li et al., 2015). Oxidative stress-induced cardiomyocyte apoptosis plays a critical role in the pathological progress of heart conditions, including coronary artery disease, cardiomyopathy, myocarditis, and hypertension (John, 2003; Mishra and Samanta, 2012). Increasing research evidence greatly demonstrated that apoptosis is one of the major mechanisms of cardiomyocytes damage following oxidative stress (Mao et al., 2014). Excessive reactive oxygen species (ROS) accumulation in a cell can lead to DNA damage, mitochondrial dysfunction, further ROS generation, cellular damage and even myocyte apoptosis (Zhu and Zuo, 2013; Li et al., 2015). Oxidative stress initiates the mitochondrial apoptotic pathway by inducing ROS overproduction and the loss of mitochondrial membrane potential, which stimulates cytochrome c release, caspase-3 activation, and subsequent poly (ADP-ribose) polymerase (PARP) cleavage, ultimately resulting in apoptosis (Ryter et al., 2007; Jia et al., 2012). Thus, the inhibition of intracellular ROS levels and prevention of cardiomyocyte apoptosis could be an effective strategies to protect oxidative stress-induced cardiomyocytes damage.

**Figure 1:**
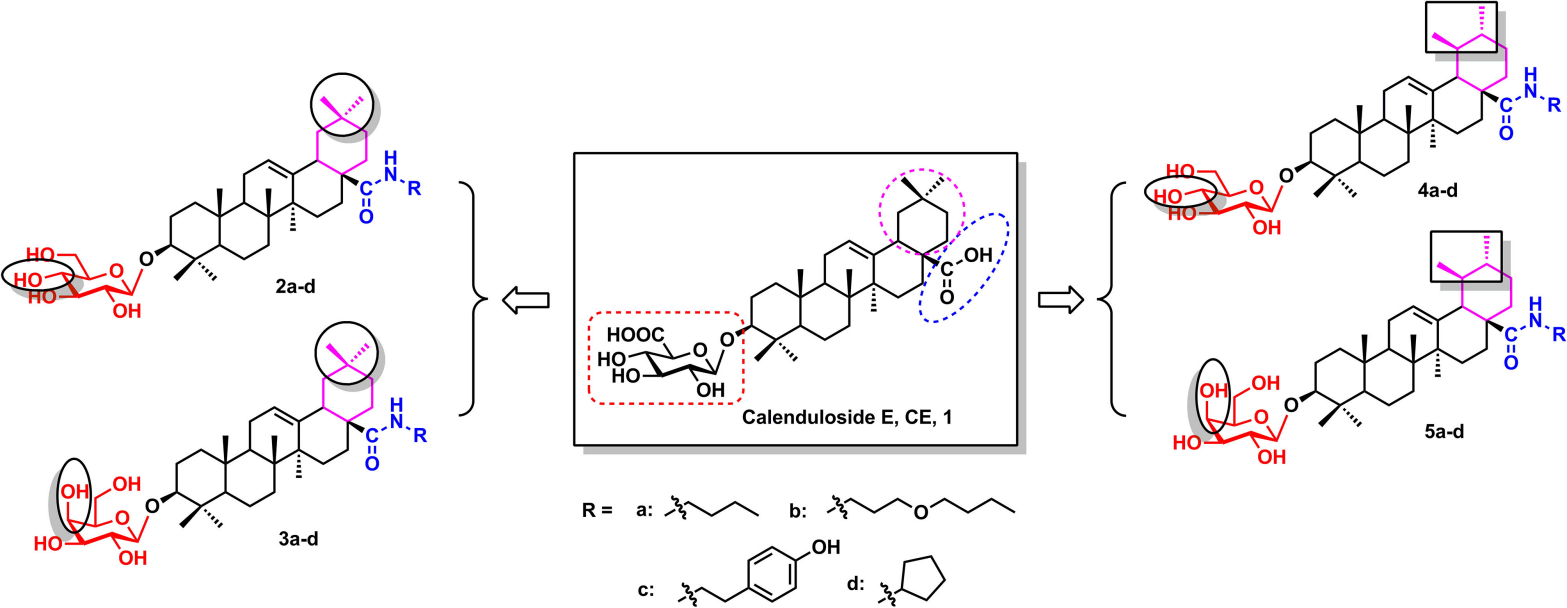
The structures of CE and its synthetic analogues.

Calenduloside E (CE, **1**, Figure 1) is one of the major natural pentacyclic triterpenoid saponins isolated from the bark and root of *Aralia elata* (Miq) Seem (AS), which is traditionally used as a tonic, antiarrhythmic, anti-arthritic, antihypertensive and anti-diabetic agent in traditional Chinese medicine (Nhiem et al., 2011; Wang et al., 2014a). The total saponins extracted from AS, which are found to be the main pharmacological active ingredients of AS, have been proved to exhibit anti-myocardial ischemic, anti-hypoxic activities, anti-oxidative as well as anti-inflammatory and anti-apoptotic capacity (Wang et al., 2003; Lee et al., 2009). We previously demonstrated that triterpenoid saponin Elatoside C and Araloside C from AS could protect the myocardial cells from ischaemia/reperfusion (IR) injury and reduce I/R-induced oxidative stress and apoptosis in cardiomyocytes (Wang et al., 2014b, 2015, 2017). We also reported that the proteomic profiling of CE targets associated with anti-apoptosis effect in endothelia cells (Tian et al., 2017). However, no literature highlighted the protective effects of CE synthetic analogues on cardiomyocytes damage, and the possible mechanisms have not yet been elucidated.

Therefore, the purpose of the current research was to synthesize novel CE analogues (**2a**–**d**, **3a**–**d**, **4a**–**d**, **5a**–**d**, Figure 1) and investigate the effects of the synthetic analogues on H9c2 cardiomyocytes subjected to H_2_O_2_-induced oxidative stress and apoptosis. The underlying mechanisms of the synthetic analogues protection were also elucidated using measuring intracellular ROS, the mitochondrial membrane potential, and the expression of apoptosis-related proteins Bcl-2, Bax, caspase-3 and PARP.

## Materials and methods

### Materials

All the reagents were used without further purification unless otherwise specified. Solvents were dried and redistilled prior to use in the usual manner. Analytical TLC was performed using silica gel HF254. Preparative column chromatography was performed with silica gel H. ^1^H and ^13^C NMR spectra were recorded on a Bruker Advance III 600 MHz spectrometer. HRMS were obtained on a Thermofisher LTQ-Obitrap XL. CE was provided from the Institute of Medicinal Plant Development (Beijing, China) (Tian et al., 2017). Cell culture products were purchased from Gibco BRL (Grand island, NY, United States). The fluorescent dye JC-1 was purchased from Sigma-Aldrich (St. Louis, MO, United States). The Annexin V/propidium iodide (PI) apoptosis detection kit was obtained from Invitrogen Corporation (Eugene, OR, United States). Caspase-3 fluorometric assay kits were acquired from BioVision (Milpitas, CA, United States). Primary antibodies against Bcl-2, Bax, Caspase-3, PARP and b-actin were obtained from Santa Cruz Biotechnology, Inc. (Santa Cruz, CA, United States).

### Chemistry

**Scheme 1:**
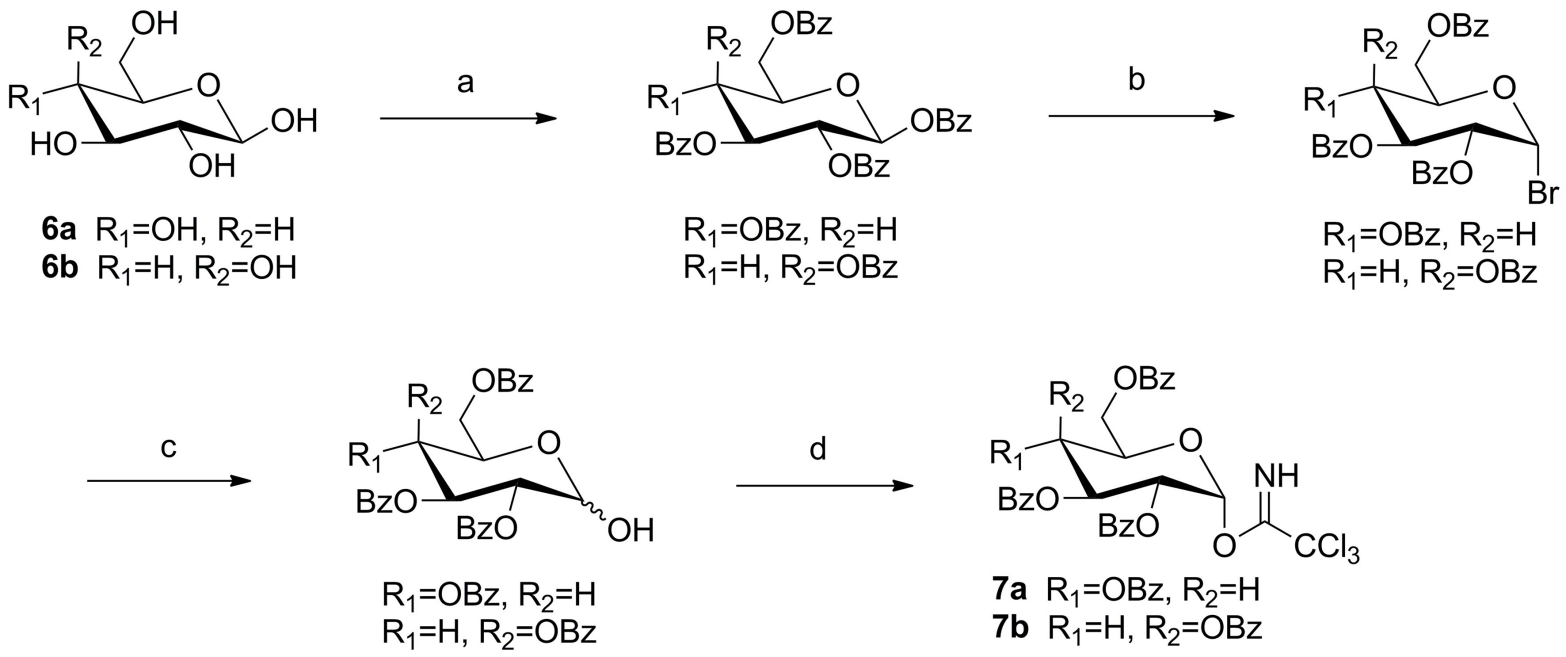
The synthesis of trichloroacetimidate sugar donors. Reagents and conditions: **(a)** BzCl, pyridine, rt, 18 h; **(b)** 33% HBr-HOAc, DCM, rt, 5 h; **(c)** Ag_2_O, acetone, H_2_O, rt, 3 h; **(d)** CCl_3_CN, K_2_CO_3_, DCM, rt, 6 h, yield: 76% (10a), 65% (10b) for four steps.

Glycosyl donor **7a** or **7b** was prepared respectively from glucose (**6a**) or galactose (**6b**), and the reaction conditions were reported previously by Schmidt and Michel (1980) (Scheme 1).

### General procedure for the synthesis of compounds 9a–b

**Scheme 2:**
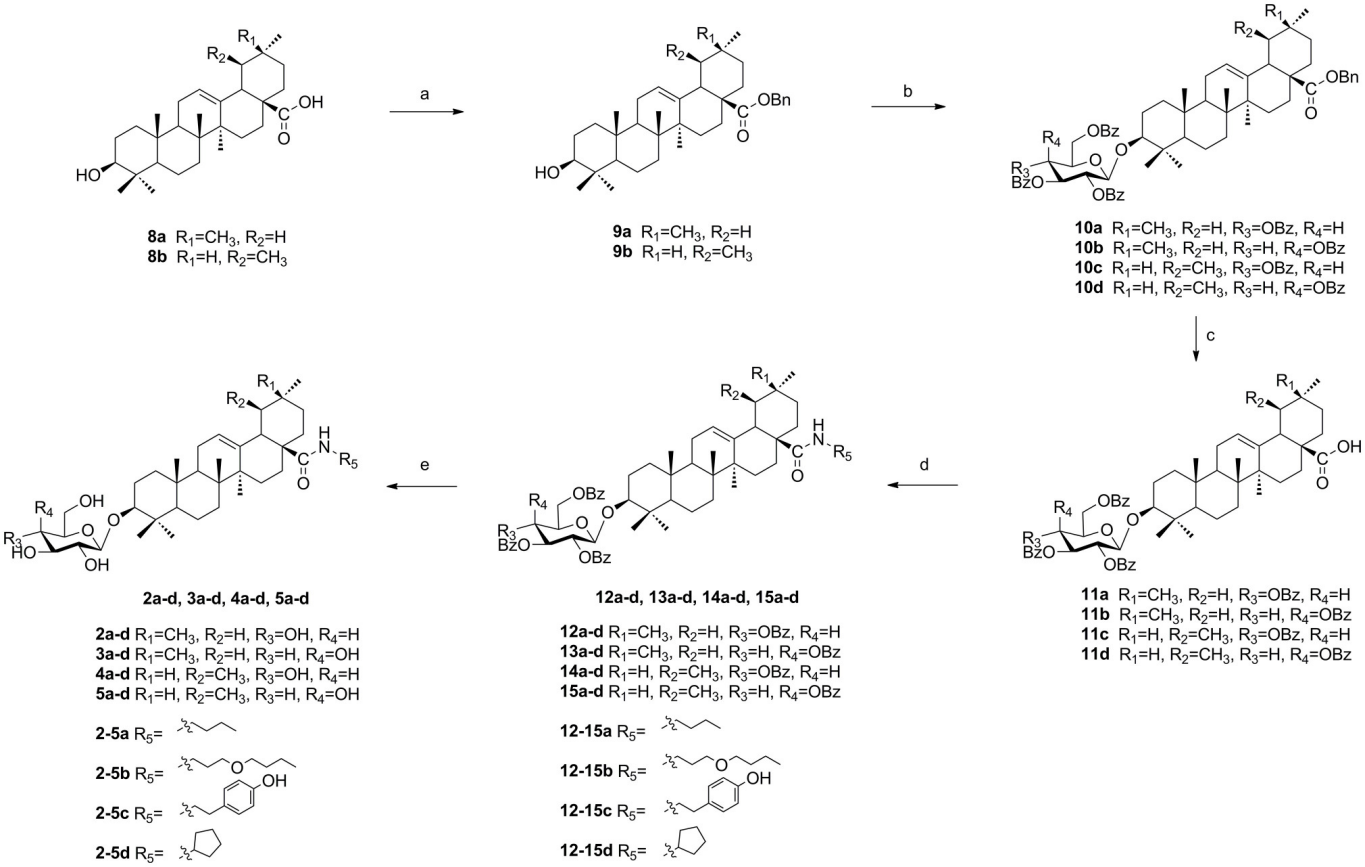
The synthesis of target compounds. Reagents and conditions: **(a)** BnBr, K_2_CO_3_, TBAB, DCM-H_2_O, rt, 18 h; **(b) 7a** or **7b**, TMSOTf, 4Å MS, DCM, rt, 2–4 h; **(c)** H_2_, Pd-C (10%), EtOAc, reflux, 4–6 h; **(d)** HOBt, EDCI, R_5_NH_2_, rt, 4-16 h; **(e)** NaOMe, MeOH, rt, 2–3 h.

To a solution of oleanolic acid **(8a)** or ursolic acid **(8b)** (10.0 g, 21.8 mmol) in dry DCM (300 mL), TBAB (0.8 g, 2.5 mmol) and K_2_CO_3_ (7.4 g, 53.6 mmol) in water (50 mL) were added, and benzyl bromide (3.2 mL, 26.8 mmol) was dropped at 0°C. Then the reaction mixture was stirred at room temperature for 18 h. Reaction was monitored by TLC. The crude mixture was separated and the water layer was extracted with DCM (3 × 100 mL). The combined organic layer was washed with 0.1 mol/L HCl aqueous solution, NaHCO_3_ saturated aqueous solution and NaCl saturated aqueous solution in sequence, and then dried over Na_2_SO_4_ and purified through column chromatography (eluent: PE-EtOAc, 8:1) to offer pure white solid **9a** (10.8 g, 91% yield) or **9b** (11.1 mg, 93% yield) (Scheme 2).

### General procedure for the synthesis of compounds 10a–d

To a solution of compound **(9a)** or **(9b)** (3.3 g, 6.0 mmol) in dry DCM (50 mL), glycosyl donor **7a** or **7b** (Schmidt and Michel, 1980) (5.8 g, 7.9 mmol) and 4Å molecular sieve 0.5 g were added and stirred at room temperature for 1 h under N_2_ air. Then lewis acid TMSOTf (60 μg, 0.3 mmol) was dropped and reacted for 2–4 h. When complete, triethylamine 1.0 mL was added to quench the reaction. Then the suspension was filtered out and the filtrate was evaporated and the crude product was subjected to column chromatography (eluent: PE-EtOAc, 10:1) to gain pure compound **10a** (5.3 g, 79% yield), **10b** (5.1 g, 75% yield), **10c** (4.7 g, 69% yield), and **10d** (4.7 g, 70% yield) as white solid. (Scheme 2).

### General procedure for the synthesis of compounds 11a–d

A mixture of **10a**–**d** (3.0 g, 2.6 mmol) and 10% Pd/C (1.5 mg) was hydrogenated at 1 atm for 4–6 h in refluxing EtOAc (30 mL). The mixture was filtered and concentrated, the residue was purified by silica gel column chromatography (eluent: PE-EtOAc, 3:1) to get pure compound **11a** (2.5 g, 92% yield), **11b** (2.5 g, 93% yield), **11c** (2.4 g, 90% yield) and **11d** (2.4 g, 91% yield) as white solid. (Scheme 2).

### General procedure for the synthesis of compounds 12a–d, 13a–d, 14a–d, 15a–d, and compounds 2a–d, 3a–d, 4a–d, 5a–d

To a solution of compound **11a**–**d** (1.0 g, 0.98 mmol) in dry DCM (15 mL), HOBt (0.2 g, 1.46 mmol) and EDCI (0.28 g, 1.46 mmol) were added and stirred at room temperature for 1 h. To this mixture, various amines (3.92 mmol) were added respectively at 0°C and the reaction mixture was stirred until its completion during 4–16 h. The solvent was washed with 0.1 mol/L HCl aqueous solution, NaHCO_3_ saturated aqueous solution and NaCl saturated aqueous solution in sequence, and then dried over Na_2_SO_4_. The suspension was filtered and the filtrate was concentrated to give compounds **12a**–**d**, **13a**–**d**, **14a**–**d**, and **15a**–**d**, which were used without further purification. To a solution of compound **12a**–**d**, **13a**–**d**, **14a**–**d**, and **15a**–**d** in MeOH/DCM (8 mL, 3:1) were added 1 mol/L NaOMe/NaOH solvent (1.6 mL). The reaction mixture was stirred for 2–3 h until its completion, after that Amberlite IR-120 was added to acidate PH 7. The suspension was filtered out and the filtrate was evaporated and purified through column chromatography (eluent: DCM-CH_3_OH, 10:1) to offer pure white solid **2a**–**d**, **3a**–**d**, **4a**–**d**, **5a**–**d** (Scheme 2).

The characterization and spectrogram of the compounds 2a–d, 3a–d, 4a–d, 5a–d were shown in Electronic Supplementary Material (ESI).

#### Biological studies

##### Cell culture and treatment

The H9c2 rat myocardial cells line was obtained from the Chinese Academy of Sciences Cell Bank (Shanghai, China) and cultured as previously described. Briefly, H9c2 cells were cultured in high glucose DMEM supplemented with 10% (v/v) fetal bovine serum, 1% penicillin/streptomycin (v/v), and 2 mM L-glutamine. The cells were maintained in a humidified incubator with 95% air and 5% CO_2_ at 37°C. The cells were subcultured after reaching 70~80% confluence (Mao et al., 2014). Three sets of experiments were performed: (1) control cells; (2) cells treated with H_2_O_2_ (450 μM) for 1 h; (3) cells pretreated with indicated concentrations (0.02, 0.1, 0.5 μg/mL) of CE analogues for 12 h, then removed the medium and treated with H_2_O_2_ (450 μM) for 1 h. For all of the experiments, the cells were plated at an appropriate density according to the experimental design and were grown for 36 h before experimentation.

##### Determination of cell viability

Cell viability was determined by the 3-(4, 5-dimethylthiazol-2-yl)-2, 5-diphenyl tetrazolium (MTT) assay as previously described (Sun et al., 2012). Briefly, H9c2 cells were plated on 96-well plates at a density of 1 × 10^4^ cells/well and incubated overnight. After designated treatment, 20 μL of MTT (5 mg/mL) was added to each well and cells were incubated at 37°C for 4 h. Then, the culture medium with MTT was abandoned and the colored formazan crystals were dissolved in 100 μL of dimethyl sulfoxide (DMSO). The absorption values were measured at 570 nm using a microplate reader (TECAN Infinite M1000, Austria). The viability of H9c2 cells in each well was presented as percentage of control cells.

### Lactate dehydrogenase (LDH) measurement

The cells were grown to confluence in 24-well plates. After drug treatment the culture supernatant was collected for analysis of LDH. The LDH leakage was determined by using commercially available kits. All the measurements were performed according to the manufacturer's protocols.

### Determination of mitochondrial transmembrane potential (ΔψM)

We used 5, 5′, 6, 6′-Tetrachloro-1, 1′, 3, 3′-tetraethylbenzimidazolyl- carbocyanine iodide (JC-1) (Sigma-Aldrich, St. Louis, USA) to analyse changes in mitochondrial transmembrane potential as previously described (Sun et al., 2012). H9c2 cells were cultured on cover slips. After treatment, the cells were washed twice with warm PBS, and incubated with JC-1 (2 μM final concentration) for 30 min in the dark, finally washed twice with PBS, images were captured using a fluorescence microscope (Leica, Germany).

### Flow cytometric detection of apoptosis

The percentage of apoptosis was measured using an Annexin V-FITC/PI apoptosis kit for flow cytometry according to the manufacturer's instructions (Invitrogen). After treatment, the cells were harvested and washed twice with cold PBS, and then incubated with 5 μL FITC-Annexin V and 1 μL PI working solution (100 μg/mL) for 15 min in the dark at room temperature. Cellular fluorescence was measured by flow cytometry analysis (FACS Calibur™, BD Biosciences, CA, USA).

### Western blot analysis

Cell lysate preparation and western blot analysis were performed as previously described (Sun et al., 2012). After treatment, H9c2 cells were harvested and lysed with cell lysis buffer containing 1% phenylmethylsulfonyl fluoride. The lysate was centrifuged for 15 min at 12,000 × *g* and 4°C to remove insoluble materials. Protein concentration was measured by the bicinchoninic acid assay using a BCA kit (Pierce Corporation, Rockford, USA). Equal amounts of protein (20 μg) from each sample were separated by 12% SDS-PAGE and transferred onto a nitrocellulose membrane (Millipore Corporation, USA). Nonspecific sites were blocked by incubating the membranes (2 h at room temperature) in 5% (w/v) non-fat milk powder in Trisbuffered saline containing 0.05% (v/v) Tween-20 (TBS-T). Thereafter, the membranes were incubated overnight at 4°C with appropriate primary antibodies. The membranes were washed with TBS-T and incubated with the appropriate secondary HRP-conjugated antibodies at 1:4,000 dilutions. Following a 30 min wash, the membranes were visualized by enhanced chemiluminescence using a Bio-Rad imaging system (Bio-Rad, Hercules, CA, USA).

### Statistical analysis

The data are expressed as mean ± SD. Comparisons were performed by Student's *t*-test or one-way ANOVA followed by Tukey's multiple comparison test with Prism 5.00 software. Statistical significance was set at *P* < 0.05. All data are the result of at least three independent experiments.

## Results

### Design and synthesis of four varieties CE analogues (2a–d, 3a–d, 4a–d, and 5a–d) based on CE

According to previous studies, the biotinconjugated CE analogue (BCEA), which maintains the active moiety of the parental compound CE, exhibits similar protective effects against ox-LDL-induced human umbilical vein endothelial cell (HUVEC) injury and identified 128 proteins related to cell survival signaling pathways as the targets (Tian et al., 2017). Based on researches of the structure-activity relationship (SAR), the amide analogues of CE at the carboxyl group did not influence the potency of the compound. In the current research, we described the design and construction of four varieties CE analogues (**2a–d**, **3a–d**, **4a–d**, **5a–d**, Figure 1) and the subsequent biological evaluation in protecting H9c2 cardiomyocytes against H_2_O_2_-induced apoptosis. First, besides the oleanane aglycone, another natural pentacyclic triterpenoid ursane which has a similar structure to oleanane was introduced to research the effect of different aglycone. Next, glucosyl and galactosyl donor were introduced to investigate the effect of glycosyl substitution type. Finally, various amide groups including n-butyl, 3-butoxypropyl, cyclopentyl and 4-hydroxyphenylethyl groups were introduced at the C-28 carboxylic moiety of the saponin scaffold to study the effect of an amide moiety on CE.

As illustrated in (Scheme 2), naturally abundant oleanolic acid **(8a)** or ursolic acid **(8b)** was treated with benzyl bromide (BnBr), potassium carbonate solution (K_2_CO_3_), and tetrabutylammonium bromide (TBAB) in dry dichloromethane (DCM) to obtain a good yield of **9a** or **9b**. The glycosyl donor **7a** or **7b** were prepared from galactose using the conditions reported by Schmidt and Michel (1980). Compound **9a** or **9b** reacted with glycosyl donor **7a** or **7b** in Lewis acidic conditions trimethylsilyl trifluoromethanesulfonate (TMSOTf) to provided compound **10a**–**d**, which was subjected severally to hydrogenation to obtain compound **11a**–**d** in the presence of catalytic amount of 10% Pd-C at atmospheric pressure. The above reaction conditions were reported in our previous paper (Tian et al., 2017). In the final two steps, four groups **12a**–**d**, **13a**–**d**, **14a**–**d**, and **15a**–**d** were attained via amidation with various amines of C-28 carboxyl group of saponin scaffold, and then followed by deprotection of glycosyl-groups in the presence of NaOMe/MeOH solution to gain final compounds **2a**–**d**, **3a**–**d**, **4a**–**d**, and **5a**–**d**.

### CE analogues alleviated H_2_O_2_-induced damage in H9c2 cells

**Figure 2:**
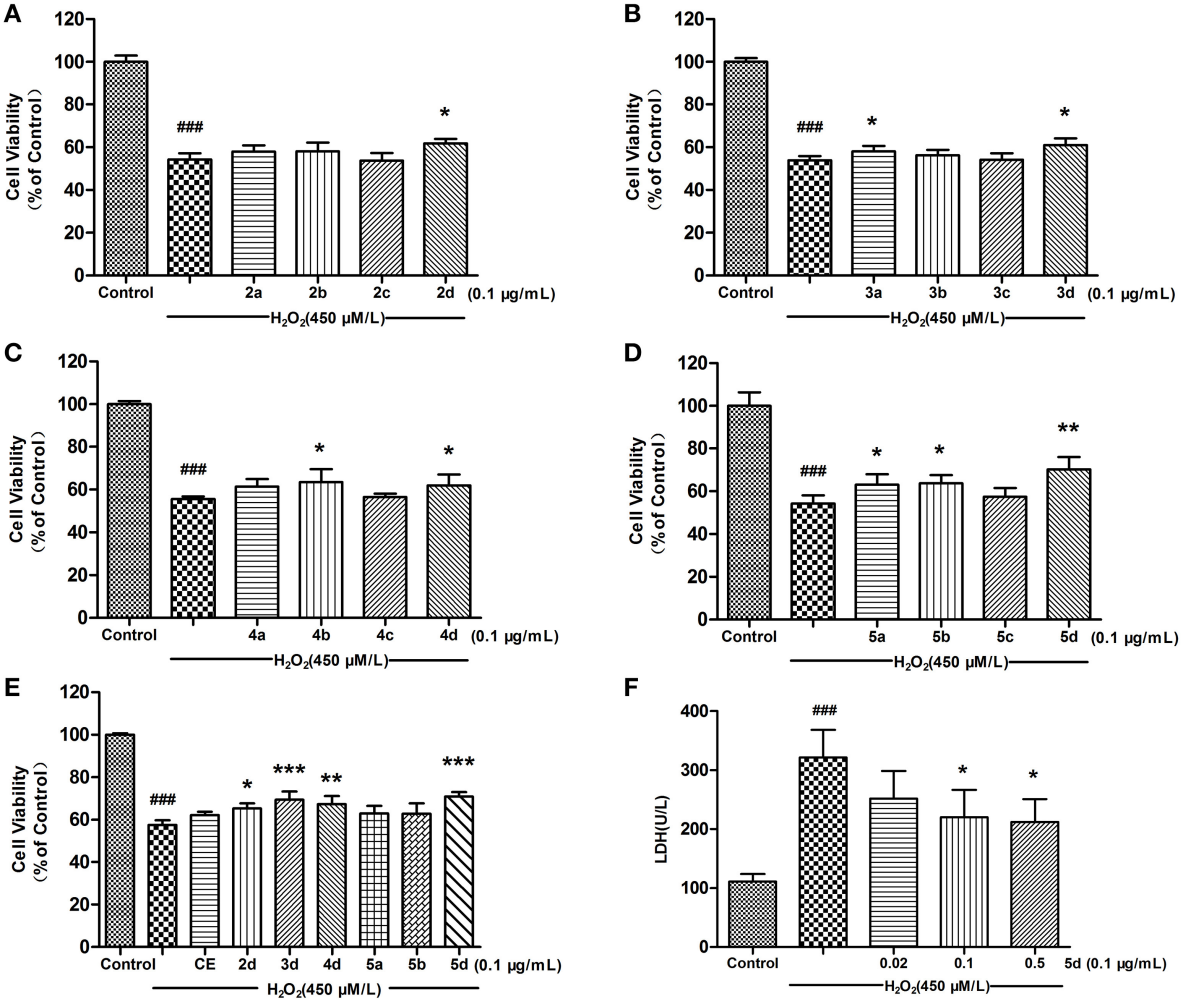
Effects of CE and CE analogues on H_2_O_2_-induced cell damage in H9c2 cells. Pre-treatment of H9c2 cells with 0.1 μg/mL concentration of CE and CE analogues for 12 h, followed by exposure to 1 h of H_2_O_2_ (450 μmol/L). Cell viability was determined using the MTT assay. **(A)** Analogues **2a**–**d**. **(B)** Analogues **3a**–**d**. **(C)** Analogues **4a**–**d**. **(D)** Analogues **5a**–**d**. **(E)** CE and CE analogues **2–5d**, **5a**, and **5b**. **(F)** The effect of analogue **5d** on the level of extracellular LDH was measured using an LDH assay kit. The data are expressed as the means ± S.D. from three independent experiments. ^###^*P* < 0.001 vs. control group; ^*^P < 0.05 vs. H_2_O_2_-treated cells; ^**^P < 0.01 vs. H_2_O_2_-treated cells; ^***^P < 0.001 vs. H_2_O_2_-treated cells.

The protective effect of CE and CE analogues against H_2_O_2_-induced cell injury was detected using 3-(4, 5-dimethylthiazol-2-yl)-2, 5-diphenyltetrazolium bromide (MTT) assay (Sun et al., 2012). As shown in Figures 2A–D, the preliminary tests of analogues **2a–d**, **3a–d**, **4a–d**, and **5a–d** at 0.1 μg/mL revealed that analogues **2–5d**, **5a**, and **5b** better increased the viability of H9c2 cells. On account of the preferable potency and typical structure, analogues **2–5d**, **5a**, and **5b** were chosen for further investigation with CE. Pretreatment of H9c2 cells with analogues **2–5d** exhibited a better protective effect than CE. The survival rate increased from 57.44% (with H_2_O_2_ treatment alone) to 65.25, 67.21, 69.42, and 70.86% after pretreatment with 0.1 μg/mL analogues **2–5d**, respectively. Among them, analogue **5d** exhibited a more potent cytoprotective effect than other compounds after pretreatment for 12 h (Figure 2E), which suggests that analogue **5d** deserves further evaluation as a potential therapeutic agent for protection against H_2_O_2_-induced H9c2 cell damage. Lactate dehydrogenase (LDH), which is secreted from cells after plasma membrane disruption, could be employed as an indicator of cell damage (Wang et al., 2010). As shown in Figure 2F, pretreatment of analogue **5d** at concentrations ranging from 0.02 to 0.5 μg/mL decreased the LDH levels in the culture medium in a concentration-dependent manner. The effects are consistent with protective effects on cell viability, as assessed using an MTT assay.

### Analogue 5d attenuated mitochondrial membrane potential (ΔΨm) disruption and H_2_O_2_-induced ROS generation in H9c2 cells

**Figure 3:**
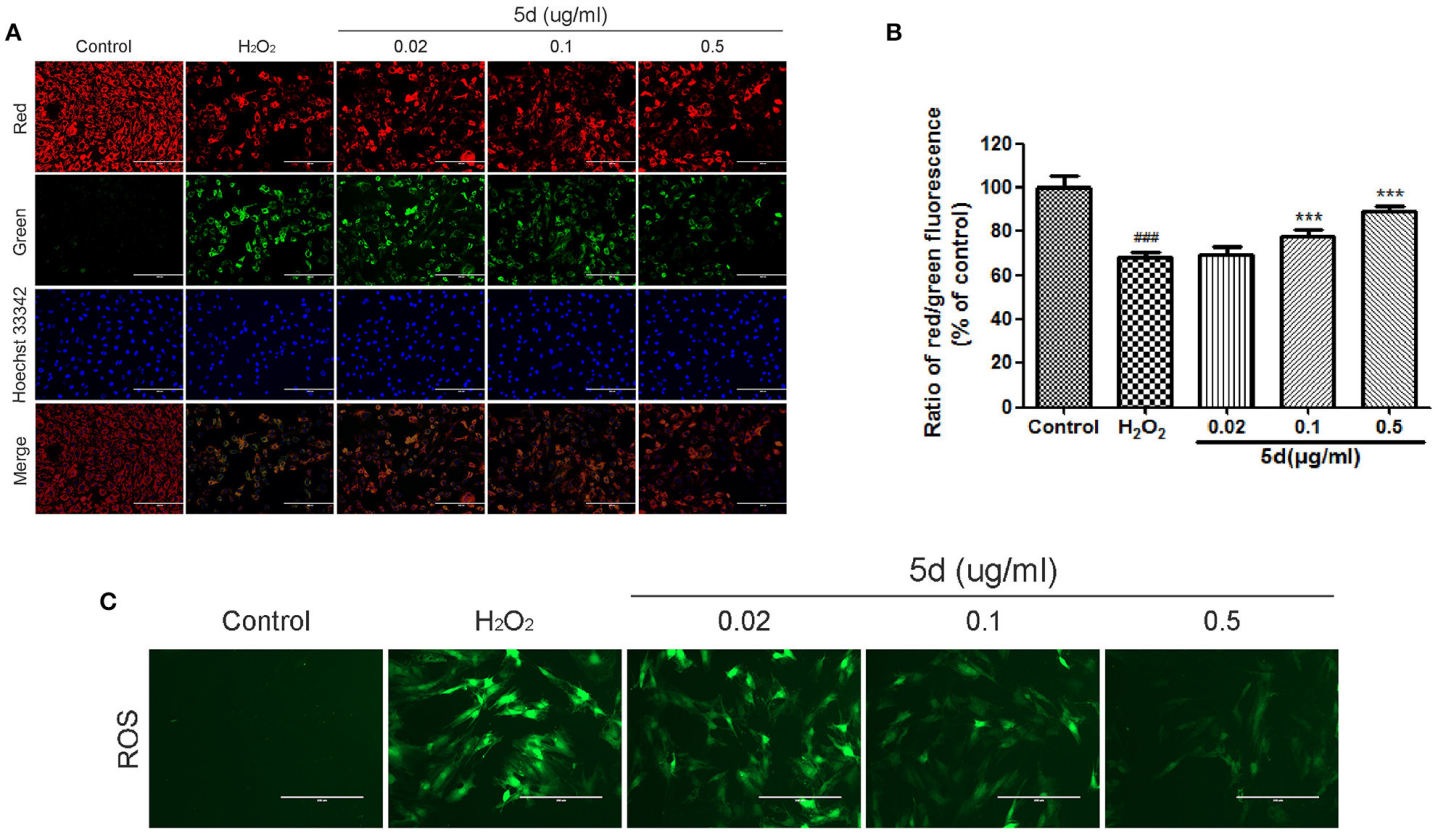
Analogue **5d** attenuated MMP decrease and attenuated H_2_O_2_-induced intracellular ROS generation. **(A)** Representative fluorescent images of JC-1 with analogue **5d** in the presence H_2_O_2_ were shown in the change of mitochondrial membrane high potential (red) and low potential (green). **(B)** The mitochondrial membrane potential was quantitatively determined with JC-1. **(C)** The cells were treated with analogue **5d** for 12 h before H_2_O_2_ exposure for 1 h, ROS fluorescence was taken photos. The data are expressed as the means ± S.D. from three independent experiments. ^###^*P* < 0.001 vs. control group; ^***^P < 0.001 vs. H_2_O_2_-treated cells.

Mitochondrial membrane potential (ΔΨm), tested by JC-1 assay, is an important parameter reflecting the function of mitochondria. JC-1 exhibits potential-dependent accumulation in mitochondria which is manifested by a fluorescence emission shift from green to red. As illustrated in Figure 3A, the treatment of H9c2 cells with H_2_O_2_ resulted in a significant decrease in the ratio of red/green fluorescence intensity, which indicated ΔΨm dissipation. However, analogue **5d** pre-treatment alleviated H_2_O_2_-induced loss of ΔΨm dose-dependently in Figure 3B. A fluorescence assay was used to determine the level of intracellular ROS, which exhibited green fluorescence under the microscope. We observed that H_2_O_2_ treatment significantly increased the H2DCFDA fluorescence compared with control cells, whereas pretreatment with analogue **5d** reduced the H_2_O_2_-induced ROS production (Figure 3C).

### Analogue 5d decreased H_2_O_2_-induced apoptosis in H9c2 cells

**Figure 4:**
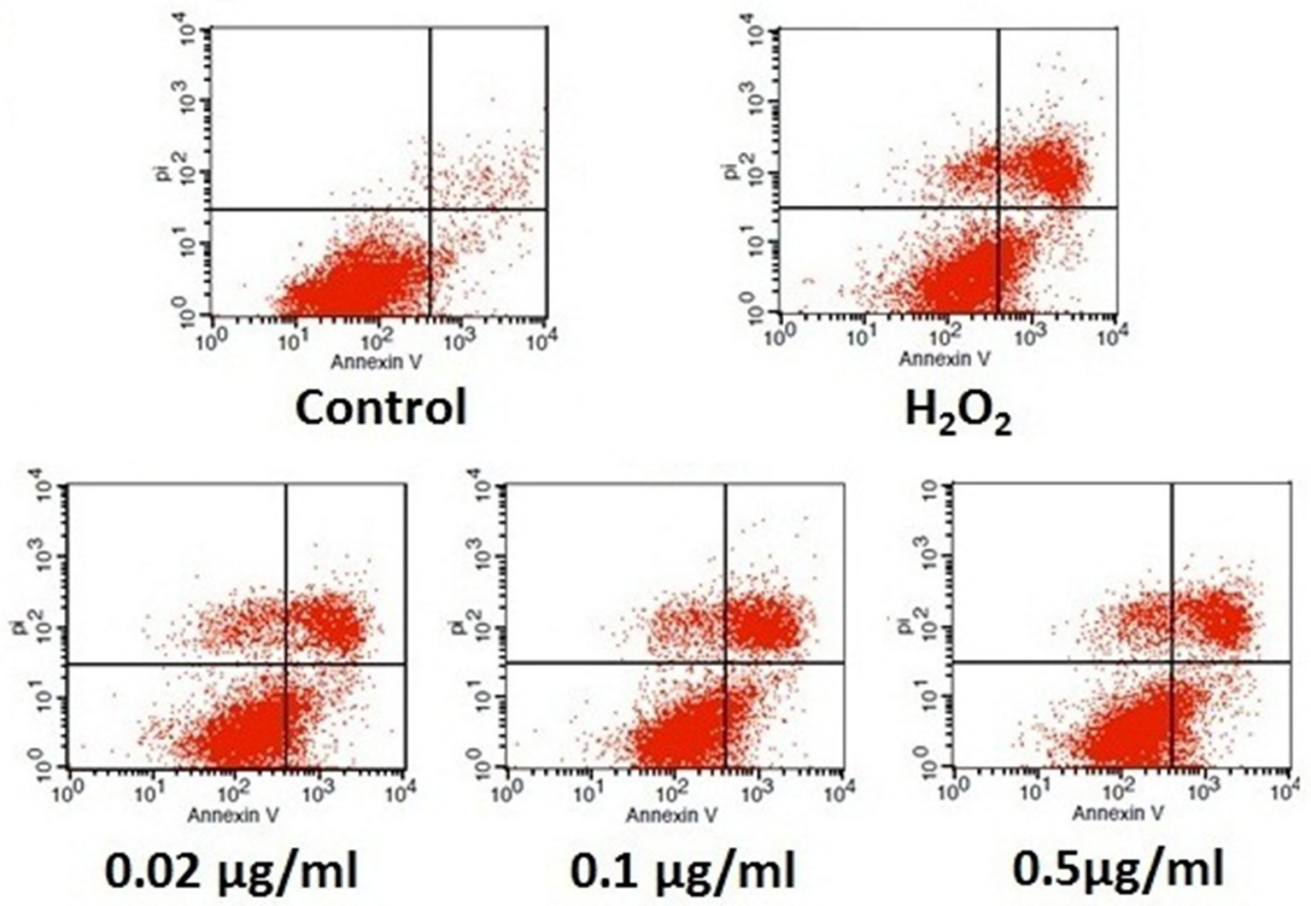
Anti-apoptotic effect of analogue **5d** on H_2_O_2_-induced damage in H9c2 cells determined by Annexin V-FITC/PI. The data are expressed as the means ± S.D. from three independent experiments.

We further confirmed the anti-apoptotic effect of analogue **5d** through the quantitative analysis of FITC-Annexin V/PI staining by flow cytometry. Annexin V can identify apoptotic cells by binding to PS exposed on the outer leaflet, whereas PI is a flourescent dye that is impermeant to live cells and apoptotic cells, but binds tightly to the nucleic acids in dead cells. As shown in Figure 4, the apoptosis rate was significantly increased in the H_2_O_2_ model group. However, pre-incubation with analogue **5d** (0.02, 0.1, 0.5 μg/mL) for 12 h prior to H_2_O_2_ exposure remarkably reversed these changes. These results suggested that analogue **5d** could rescue H9c2 cells from H_2_O_2_-induced apoptotic death.

### Analogue 5d modulated apoptosis-related protein activity and expression in H_2_O_2_-treated H9c2 cells

**Figure 5:**
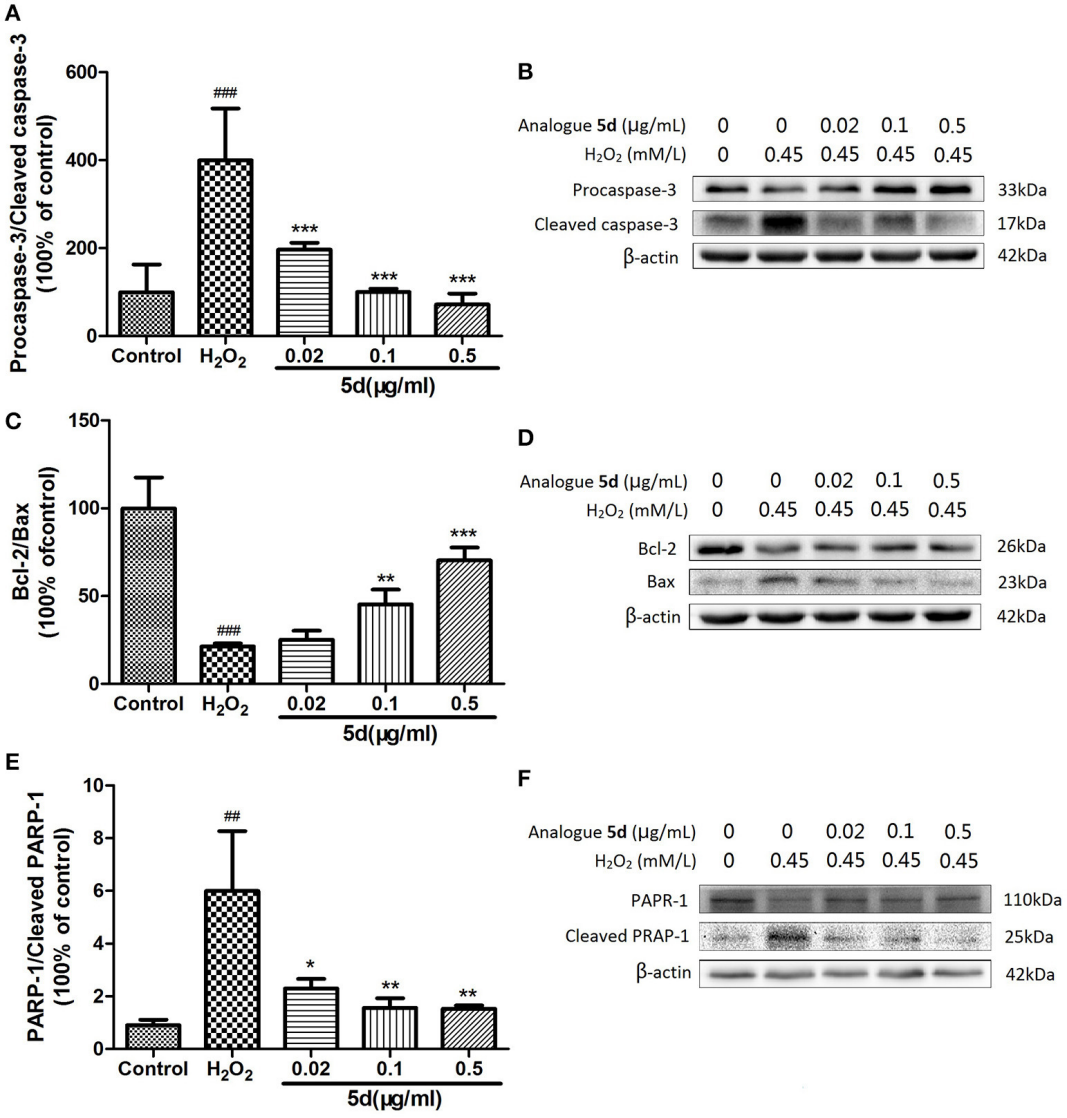
Effects of analogue **5d** on apoptotic-related protein expression and activity. Proteins related to apoptotic signaling were analyzed using Western blotting. **(A)** Procaspase-3/Cleaved caspase-3 ratio; **(B)** Western blot analysis of Procaspase-3 and Cleaved caspase-3; **(C)** Bcl-2/Bax ratio; **(D)** Western blot analysis of Bcl-2 and Bax; **(E)** PARP-1/Cleaved PARP-1 ratio; **(F)** Western blot analysis of PARP-1 and Cleaved PARP-1; β-Actin expression was examined as the protein loading control. The data are presented as the means ± S.D. from three independent experiments. ^##^*P* < 0.01 vs. control group; ^###^*P* < 0.001 vs. control group; ^*^P < 0.05 vs. H_2_O_2_-treated cells; ^**^P < 0.01 vs. H_2_O_2_-treated cells; ^***^P < 0.001 vs. H_2_O_2_-treated cells.

To further understand the mechanism of analogue **5d** treatment on the regulation of cell apoptosis, we measured the effects of analogue **5d** on the expression of apoptosis-related protein expression using Western blot analysis. As shown in Figures 5A,B, the H_2_O_2_ group showed a decrease in expression of the anti-apoptotic protein Bcl-2 and an increase in expression of the pro-apoptotic protein Bax. Pretreatment with analogue **5d** (0.5 μg/mL) increased the Bcl-2/Bax expression ratio compared with the H_2_O_2_ group (70.37 ± 7.37 vs. 21.30 ± 1.80, respectively). As shown in Figures 5C–F, the amount of Procaspase-3 and PARP-1 on H_2_O_2_-induced injury in H9c2 cells were markedly decreased, which were the signs of caspase activation and PARP-1 cleavage that lead to apoptosis. However, analogue **5d** pretreatment decreased Procaspase-3/Cleaved caspase-3 and PARP-1/Cleaved PARP-1 expression ratio dose-dependently compared with the H_2_O_2_ group. Taken together, these results indicated that analogue **5d** inhibited apoptosis markedly on H_2_O_2_-induced injury in H9c2 cells.

## Discussion

In the present research, we first utilized chemical semisynthesis and biological evaluation approaches to investigate protective effects of 16 Calenduloside E analogues and analyzed their SAR. Among them, the analogue **5d** exhibited more potent activity both than other analogues and CE on H_2_O_2_-induced H9c2 cell damage. Moreover, further evaluation with JC-1 staining assays, fluorescence assay and flow cytometry confirmed the anti-apoptotic effect of analogue **5d**. We ultimately focused on apoptosis-related protein caspase-3, Bcl-2/Bax and PARP-1 using the western blotting strategy, and these above results suggested that the analogue **5d** might exert protective effects through modulating the expression of these apoptosis-related proteins.

The design and synthesis of potential analogues represents a major challenge in structural modification of natural product CE. In our previous study, the introduction of a substituent at the C-28 position of CE maintained its protective effects. Based on the results of previous research, amide derivatives of CE containing ursane and galactoside scaffolds maintained similar activity to the parental compound CE (Tian et al., 2017). In the current study, *N*-normal-butylamide, *N*-3-butoxypropylamide, *N*-cyclopentylamide and *N*-4-hydroxyphenylethyl- amide **a–d** series were chosen as CE analogues in which the amide groups were introduced using an acylation reaction on nitrogen-atoms. On account of the scaffolds types of aglycones and glycosyl donors, we designed and synthesized sixteen derivatives and divided into four groups (**2a–d**, **3a–d**, **4a–d**, and **5a–d**, Figure 1) to evaluate and compare their protective activity on H_2_O_2_-induced damage in H9c2 Cells.

Enhanced oxidative injury after various stimuli has been confirmed to be an original event in the development of cardiovascular diseases. Hydrogen peroxide (H_2_O_2_) is extensively referred to as one of the major oxidative stimuli in anti-oxidative research, and rat myocardial cells (H9c2 cells) are commonly accepted as an experimental model in the study of the mechanisms involved in the pathogenesis of cardiovascular diseases (Pan et al., 2011). According to the results of the MTT assay, some analogues exhibited promising protective effects against H_2_O_2_-induced damage in H9c2 Cells. Among them, serie **d** was found to be more potent than other series at 0.1 μg/mL, and analogues **2d**, **3d**, **4d**, and **5d** increased cell viability by 7.49, 7.11, 6.41, and 16%, compared to control respectively, Figures 2A–D). As the preferable effects on MTT assays, analogues **2–5d**, **5a**, and **5b** were chosen for further SAR studies with CE. The preliminary SARs suggested that analogues **2–5d** which coupled with cyclopentyl substituents, could increase H9c2 cells viability better than CE. It was also noted that, among these **2–5d** compounds, the amide derivative **5d** containing ursane and galactoside scaffolds could be the best analogue to improve cell viability on this H9c2 cell line (e.g., **5d** vs. **2–4d**, Figure 2E). Given the above, the analogue **5d**, which possessed the best protective potency in these synthetic analogues of CE, was elected for further mechanistic investigation.

Multiple studies have demonstrated that mitochondrial apoptotic pathway is considered crucial in myocardial apoptosis induced by H_2_O_2_ (Mao et al., 2014; Tricarico et al., 2015). Excessive ROS damages various biomolecules via lipid peroxidation, protein oxidation, and DNA damage, thereby causing ΔΨm collapsing, caspase-3 activation and cell apoptosis (Zhu and Zuo, 2013). In accordance with a previous study (Sun et al., 2011; Yu et al., 2016), our present study demonstrated that H_2_O_2_ exposure remarkably increased ROS production and resulted in the disruption of ΔΨm in H9c2 cells. However, analogue **5d** pretreatment significantly decreased the level of intracellular ROS production and inhibited the collapse of ΔΨm (Figure 3). Our data demonstrate that the protective effect of analogue **5d** on H_2_O_2_-induced injury was probably due to the stabilization of mitochondrial dysfunction. Moreover, we also confirmed that analogue **5d** protects against apoptosis using Annexin V/PI staining (Figure 4). Based on these results, analogue **5d** effectively inhibits H_2_O_2_-induced apoptosis and provides further evidence supporting its anti-apoptotic role.

Bcl-2 and Bax are the members of Bcl-2 family that critical for the regulation of apoptosis. Bcl-2 protein is known to promote cell survival as well as to suppress cell death, whereas Bax is a pro-apoptotic protein that promotes or accelerates cell death (Kaushal et al., 2004). Caspases are a family of aspartate-specific cysteine proteases that play key roles in regulating apoptosis induced by different stimuli, including oxidative stress. Caspase-3 is the key executioner of apoptosis, regulating factors involved in DNA degradation, chromatin condensation, and nuclear fragmentation (Earnshaw et al., 1999). Active executive protein cleaved caspase-3 can further cleave downstream substrates involved in apoptotic process, such as PARP, resulting in cell apoptosis (von Harsdorf et al., 1999; Clerk et al., 2003). In the current study, pretreatment of analogue **5d** could significantly increase Bcl-2/Bax expression ratio, inhibit activation of caspase-3 as well as cleavage of PARP-1 (Figure 5). Therefore, the mechanism by which analogue **5d** suppressed cell apoptosis on H_2_O_2_-induced injury in H9c2 cells was possibly through the regulation in the intrinsic mitochondria-mediated pathway such as Bcl-2 and caspase family-related signals.

## Conclusion

The results of the present research indicated that CE anologue **5d** exerts a profound cardioprotective effect against H_2_O_2_-induced injury for H9c2 cardiomyocytes through suppression of oxidative stress and apoptosis and that anti-apoptotic mechanism contributes to the cardioprotective effect of anologues, at least in part, through the intrinsic mitochondria-mediated pathway such as Bcl-2 and caspase family-related signals. Further studies are in progress as the promising results demonstrate that these CE anologues merit further investigation as potential cardiovascular protective agents.

## Author contributions

X-DX, G-BS, and X-BS conducted the study. YT designed the detailed experiments, performed the study, and collected and analyzed data. Y-YD, HS, MW, Z-HS, B-QW and DD took part in the cell experiments in this study. All authors commented the study and approved the final manuscript.

### Conflict of interest statement

The authors declare that the research was conducted in the absence of any commercial or financial relationships that could be construed as a potential conflict of interest.
