# Supplementary material for: Calenduloside E Analogues Protecting H9c2 Cardiomyocytes Against H2O2-Induced Apoptosis: Design, Synthesis and Biological Evaluation
Source: Front Pharmacol. 2017 Nov 23;8:862. doi: 10.3389/fphar.2017.00862 (PMC5703861; doi:10.3389/fphar.2017.00862)
Supplement: Supplementary file 1 [file Image1.PDF]

Calenduloside E Analogues Protecting H9c2 Cardiomyocytes against H_2O_2-induced Apoptosis: Design, Synthesis and Biological evaluation

Yu Tian,[a#] Yu-Yang Du,[a#] Hai Shang,[a] Min Wang,[a] Zhong-Hao Sun,[a] Bao-Qi Wang,[a, b] Di Deng,[a, b] Shan Wang,[a] Xu-Dong Xu,[a]* Gui-Bo Sun,[a]* and Xiao-Bo Sun[a]*

[a] Beijing Key Laboratory of Innovative Drug Discovery of Traditional Chinese Medicine (Natural Medicine) and Translational Medicine; Key Laboratory of Bioactive Substances and Resources Utilization of Chinese Herbal Medicine, Ministry of Education; Key Laboratory of Efficacy Evaluation of Chinese Medicine against Glycolipid Metabolic Disorders, State Administration of Traditional Chinese Medicine; Zhong guan cun Open Laboratory of the Research and Development of Natural Medicine and Health Products; Institute of Medicinal Plant Development, Chinese Academy of Medical Sciences & Peking Union Medical College, Beijing, 100193, P. R. China.

[b] Harbin University of Commerce, Harbin, 150076, Heilongjiang, P. R. China.

[#]These authors contributed equally to this work.

*Correspondence authors. Address: Institute of Medicinal Plant Development, Chinese Academy of Medical Sciences and Peking Union Medical College, No. 151, Malianwa North Road, Haidian District, Beijing 100193, PR China. Tel: +86-010-57833013; Fax: +86-010-57833013.

E-mail addresses: xdxu@implad.ac.cn (Xu-dong Xu), gbsun@implad.ac.cn (Gui-bo Sun), sunsubmit@163.com (Xiao-bo Sun).

The characterization of the compounds 2a−d, 3a−d, 4a−d, 5a−d

28-N-normal-Butyl oleanolic amide 3-O-β-D-glucopyranoside ***2a***

White solid, 84% yield; $[\alpha]^{25}_{D}$: +47.25 (c 0.13, MeOH); ^{1}H-NMR (600 MHz, pyridine-d_5) δ: 7.28 (t, J = 5.6 Hz, 1H, N-H), 5.47 (t, J = 3.2 Hz, 1H, H-12), 4.96 (d, J = 7.8 Hz, 1H, H-1′), 4.61 (d, J = 11.5 Hz, 1H, Glu-H), 4.44 (dd, J = 11.5 Hz, 5.3 Hz, 1H, Glu-H), 4.30−4.24 (m, 2H, Glu-H), 4.08−4.03 (m, 2H, Glu-H), 3.55−3.49 (m, 1H, H-31-1), 3.42 (dd, J = 11.8 Hz, 4.3 Hz, 1H, H-3), 3.37−3.32 (m, 1H, H-31-2), 3.11 (dd, J = 13.4 Hz, 3.9 Hz, 1H, H-18), 1.35 (s, 3H, CH_3), 1.30 (s, 3H, CH_3), 1.04 (s, 3H, CH_3), 0.98 (s, 3H, CH_3), 0.96 (s, 3H, CH_3), 0.94 (s, 3H, CH_3), 0.90 (s, 3H, CH_3), 0.85 (t, J = 7.3 Hz, 3H, H-34); ^{13}C-NMR (150 MHz, pyridine-d_5) δ: 177.3, 145.0, 122.7, 106.8, 88.8, 78.7, 78.2, 75.8, 71.9, 63.1, 55.8, 47.9, 46.8, 46.4, 42.2, 42.0, 39.8, 39.6, 39.5, 38.7, 37.0, 34.4, 33.8, 33.2, 33.1, 32.2, 30.9, 28.2, 27.9, 26.5, 26.1, 23.8 (C×2), 23.7, 20.5, 18.5, 17.5, 17.0, 15.5, 13.9; HRMS (ESI): Calcd for $[M + H]^+$ $C_{40}H_{68}NO_7$: 674.4996, found 674.4988.

28-N-Butoxypropyl oleanolic amide 3-O-β-D-glucopyranoside ***2b***

White solid, 85% yield; $[\alpha]^{25}_{D}$: +35.25 (c 0.13, MeOH); ^{1}H-NMR (600 MHz, pyridine-d_5) δ: 7.37 (t, J = 5.5 Hz, 1H, N-H), 5.46 (t, J = 3.3 Hz, 1H, H-12), 4.96 (d, J = 7.8 Hz, 1H, H-1′), 4.61 (dd, J = 11.6 Hz, 2.5 Hz, 1H, Glu-H), 4.43 (dd, J = 11.7 Hz, 5.4 Hz, 1H, Glu-H), 4.29−4.23 (m, 2H, Glu-H), 4.08−4.02 (m, 2H, Glu-H), 3.72−3.66 (m, 1H, H-31-1), 3.54−3.48 (m, 3H, H-31-2, H-33), 3.42 (dd, J = 11.7 Hz, 4.4 Hz, 1H, H-3), 3.36 (t, J = 6.6 Hz, 1H, H-34), 3.10 (dd, J = 13.2 Hz, 4.2 Hz, 1H, H-18), 1.35 (s, 3H, CH_3), 1.30 (s, 3H, CH_3), 1.04 (s, 3H, CH_3), 0.97 (s, 6H, 2×CH_3), 0.95 (s, 3H, CH_3), 0.90 (s, 3H, CH_3), 0.86 (t, J = 7.5 Hz, 3H, H-37); ^{13}C-NMR (150 MHz, pyridine-d_5) δ: 177.3, 144.9, 122.7, 106.8, 88.8, 78.7, 78.2, 75.8, 71.9, 70.8, 69.4, 63.1, 55.8, 47.9, 46.8, 46.3, 42.2, 42.0, 39.8, 39.5, 38.7, 38.0, 37.0, 34.4, 33.8, 33.1, 33.0, 32.1, 30.9, 30.3, 28.2, 27.9 26.5, 26.1, 23.8, 23.7 (C×2), 19.6, 18.4, 17.5, 17.0, 15.4, 14.0; HRMS (ESI): Calcd for $[M + Na]^+$ $C_{43}H_{73}NNaO_8$: 754.5234, found 754.5230.

28-N-(4'-Hydroxyphenylethyl) oleanolic amide 3-O-β-D-glucopyranoside ***2c***

White solid, 71% yield; $[\alpha]^{25}_{D}$: +57.00 (c 0.13, MeOH); ^{1}H-NMR (600 MHz, pyridine-d_5) δ: 7.25−7.23 (m, 2H, Ph-H), 7.18−7.17 (m, 2H, Ph-H), 7.10 (t, J = 5.6 Hz, 1H, N-H), 5.32 (t, J = 3.2 Hz, 1H, H-12), 4.96 (d, J = 7.8 Hz, 1H, H-1′), 4.61 (dd, J = 11.6 Hz, 2.3 Hz, 1H, Glu-H), 4.44 (dd, J = 11.7 Hz, 5.5 Hz, 1H, Glu-H), 4.29−4.23 (m, 2H, Glu-H), 4.08−4.02 (m, 2H, Glu-H), 3.98−3.93 (m, 1H, H-31-1), 3.51−3.46 (m, 1H, H-31-2), 3.41 (dd, J = 11.8 Hz, 4.2 Hz, 1H, H-3), 2.98−2.94 (m, 2H, H-32-1, H-18), 2.88−2.84 (m, 1H, H-32-2), 1.34 (s, 3H, CH_3), 1.27 (s, 3H, CH_3), 1.04 (s, 3H, CH_3), 0.96 (s, 3H, CH_3), 0.93 (s, 3H, CH_3), 0.89 (s, 3H, CH_3), 0.87 (s, 3H, CH_3); ^{13}C-NMR (150 MHz, pyridine-d_5) δ: 177.4, 157.5, 144.7, 130.3 (C×2), 123.0, 116.3, 106.8, 88.9, 78.7, 78.2, 75.8, 71.9, 63.1, 55.8, 47.9, 46.9, 46.4, 42.1, 42.0, 41.6, 39.7, 39.5, 38.7, 36.9, 35.4, 34.4, 33.5, 33.1, 32.9, 30.8, 28.2, 27.8, 26.5, 26.0, 23.9, 23.7, 23.6, 18.4, 17.3, 17.0, 15.5; HRMS (ESI): Calcd for $[M + Na]^+$ $C_{44}H_{67}NNaO_8$: 760.4764, found 760.4750.

28-N-Cyclopentyl oleanolic amide 3-O-β-D-glucopyranoside ***2d***

White solid, 87% yield; $[\alpha]^{25}_{D}$: +24.00 (c 0.13, MeOH); ^{1}H-NMR (600 MHz, pyridine-d_5) δ: 6.75 (d, J = 6.8 Hz, 1H, N-H), 5.48 (t, J = 3.4 Hz, 1H, H-12), 4.96 (d, J = 7.8 Hz, 1H, H-1′), 4.61 (dd, J = 11.6 Hz, 2.6 Hz, 1H, Glu-H), 4.48−4.42 (m, 2H, H-31, Glu-H), 4.29−4.23 (m, 2H, Glu-H), 4.08−4.02 (m, 2H, Glu-H), 3.42 (dd, J = 11.8 Hz, 4.3 Hz, 1H, H-3), 3.06 (dd, J = 13.4 Hz, 4.0 Hz, 1H, H-18), 1.35 (s, 3H, CH_3), 1.30 (s, 3H, CH_3), 1.05 (s, 3H, CH_3), 0.99 (s, 3H, CH_3), 0.94 (s, 3H, CH_3), 0.93 (s, 3H, CH_3), 0.92 (s, 3H, CH_3); ^{13}C-NMR (150 MHz, pyridine-d_5) δ: 177.0, 145.0, 122.7, 106.8, 88.8, 78.7, 78.2, 75.8, 71.9, 63.1, 55.8, 51.6, 47.9, 46.8, 46.2, 42.3, 42.1, 39.8, 39.5, 38.8, 37.0, 34.4, 33.6, 33.5, 33.1 (C×2), 32.5, 30.8, 28.2, 27.8, 26.5, 26.0, 24.2, 24.1, 23.8, 23.7 (C×2), 18.5, 17.6, 17.0, 15.5; HRMS (ESI): Calcd for $[M + Na]^+$ $C_{41}H_{67}NNaO_7$: 708.4815, found 708.4811.

28-N-normal-Butyl oleanolic amide 3-O-β-D-galactopyranoside ***3a***

White solid, 86% yield; $[\alpha]_D^{25}$: +45.00 (c 0.13, MeOH); ^{1}H-NMR (600 MHz, pyridine-d_5) δ: 7.26 (t, J = 5.7 Hz, 1H, N-H), 5.47 (t, J = 3.4 Hz, 1H, H-12), 4.88 (d, J = 7.6 Hz, 1H, H-1′), 4.61−4.60 (m, 1H, Gal-H), 4.52−4.46 (3H, m, Gal-H), 4.19 (dd, J = 3.4 Hz, 9.6 Hz, 1H, Gal-H), 4.14 (t, J = 5.9 Hz, 1H, Gal-H), 3.55−3.49 (m, 1H, H-31-1), 3.42 (dd, J = 11.8 Hz, 4.4 Hz, 1H, H-3), 3.37−3.31 (m, 1H, H-31-2), 3.11 (dd, J = 13.3 Hz, 4.1 Hz, 1H, H-18), 1.34 (s, 3H, CH_3), 1.31 (s, 3H, CH_3), 1.01 (s, 3H, CH_3), 0.98 (s, 3H, CH_3), 0.96 (s, 3H, CH_3), 0.94 (s, 3H, CH_3), 0.91 (s, 3H, CH_3), 0.85 (t, J = 7.4 Hz, 3H, H-34); ^{13}C-NMR (150 MHz, pyridine-d_5) δ: 177.3, 145.0, 122.7, 107.4, 88.7, 76.8, 75.4, 73.2, 70.3, 62.4, 55.8, 47.9, 46.8, 46.4, 42.2, 42.0, 39.8, 39.6, 39.5, 38.8, 37.0, 34.4, 33.8, 33.2, 33.1, 32.2, 30.9, 28.2, 27.9, 26.6, 26.1, 23.8 (C×2), 23.7, 20.5, 18.5, 17.5, 16.9, 15.5, 13.9; HRMS (ESI): Calcd for $[M + H]^+$ $C_{40}H_{68}NO_7$: 674.4996, found 674.4987.

28-N-Butoxypropyl oleanolic amide 3-O-β-D-galactopyranoside ***3b***

White solid, 85% yield; $[\alpha]_D^{25}$: +46.50 (c 0.13, MeOH); ^{1}H-NMR (600 MHz, pyridine-d_5) δ: 7.36 (t, J = 5.5 Hz, 1H, N-H), 5.46 (t, J = 3.2 Hz, 1H, H-12), 4.88 (d, J = 7.7 Hz, 1H, H-1′), 4.61−4.60 (m, 1H, Gal-H), 4.52−4.46 (m, 3H, Gal-H), 4.19 (dd, J = 3.3 Hz, 9.5 Hz, 1H, Gal-H), 4.14 (t, J = 6.0 Hz, 1H, Gal-H), 3.72−3.66 (m, 1H, H-31-1), 3.54−3.48 (m, 3H, H-31-2, H-33), 3.42 (dd, J = 11.8 Hz, 4.4 Hz, 1H, H-3), 3.36 (t, J = 6.7 Hz, 1H, H-34), 3.10 (dd, J = 13.3 Hz, 3.9 Hz, 1H, H-18), 1.34 (s, 3H, CH_3), 1.31 (s, 3H, CH_3), 1.01 (s, 3H, CH_3), 0.97 (s, 6H, 2×CH_3), 0.95 (s, 3H, CH_3), 0.91 (s, 3H, CH_3), 0.86 (t, J = 7.4 Hz, 3H, H-37); ^{13}C-NMR (150 MHz, pyridine-d_5) δ: 177.3, 144.9, 122.7, 107.4, 88.7, 76.8, 75.4, 73.2, 70.8, 70.3, 69.4, 62.5, 55.8, 47.9, 46.8, 46.4, 42.2, 42.0, 39.8, 39.5, 38.8, 38.0, 37.0, 34.4, 33.8, 33.2, 33.1, 32.1, 30.9, 30.3, 28.2, 27.9, 26.6, 26.1, 23.8, 23.7 (C×2), 19.6, 18.5, 17.5, 16.9, 15.5, 14.0; HRMS (ESI): Calcd for $[M + Na]^+$ $C_{43}H_{73}NNaO_8$: 754.5234, found 754.5234.

28-N-(4'-Hydroxyphenylethyl) oleanolic amide 3-O-β-D-galactopyranoside ***3c***

White solid, 69% yield; $[\alpha]_D^{25}$: +35.25 (c 0.13, MeOH); ^{1}H-NMR (600 MHz, pyridine-d_5) δ: 7.25−7.23 (m, 2H, Ph-H), 7.18−7.17 (m, 2H, Ph-H), 7.11 (t, *J* = 5.6 Hz, 1H, N-H), 5.32 (t, *J* = 3.2 Hz, 1H, H-12), 4.88 (d, *J* = 7.7 Hz, 1H, H-1′), 4.61−4.60 (m, 1H, Gal-H), 4.53−4.46 (3H, m, Gal-H), 4.19 (dd, *J* = 3.4 Hz, 9.5 Hz, 1H, Gal-H), 4.14 (t, *J* = 6.1 Hz, 1H, Gal-H), 3.98−3.93 (m, 1H, H-31-1), 3.51−3.46 (m, 1H, H-31-2), 3.41 (dd, *J* = 11.7 Hz, 4.4 Hz, 1H, H-3), 2.98−2.94 (m, 2H, H-32-1, H-18), 2.89−2.84 (m, 1H, H-32-2), 1.33 (s, 3H, CH_3), 1.27 (s, 3H, CH_3), 1.00 (s, 3H, CH_3), 0.96 (s, 3H, CH_3), 0.94 (s, 3H, CH_3), 0.90 (s, 3H, CH_3), 0.87 (s, 3H, CH_3); ^{13}C-NMR (150 MHz, pyridine-d_5) δ: 177.4, 157.5, 144.7, 130.3 (C×2), 123.0, 116.3, 107.4, 88.7, 76.7, 75.4, 73.2, 70.3, 62.4, 55.8, 47.9, 46.9, 46.4, 42.1, 42.0, 41.6, 39.7, 39.5, 38.7, 36.9, 35.4, 34.4, 33.5, 33.1, 32.9, 30.8, 28.2, 27.8, 26.6, 26.0, 23.9, 23.7, 23.6, 18.4, 17.3, 16.9, 15.5; HRMS (ESI): Calcd for $[M + Na]^+$ $C_{44}H_{67}NNaO_8$: 760.4764, found 760.4764.

*28-N-Cyclopentyl oleanolic amide 3-O-β-D-galactopyranoside **3d***

White solid, 81% yield; $[\alpha]_D^{25}$: +31.50 (c 0.13, MeOH); ^{1}H-NMR (600 MHz, pyridine-d_5) δ: 6.75 (d, *J* = 6.7 Hz, 1H, N-H), 5.48 (t, *J* = 3.5 Hz, 1H, H-12), 4.88 (d, *J* = 7.6 Hz, 1H, H-1′), 4.61−4.60 (m, 1H, Gal-H), 4.52−4.44 (m, 4H, H-31, Gal-H), 4.19 (dd, *J* = 3.3 Hz, 9.5 Hz, 1H, Gal-H), 4.14 (t, *J* = 5.9 Hz, 1H, Gal-H), 3.42 (dd, *J* = 11.7 Hz, 4.3 Hz, 1H, H-3), 3.06 (dd, *J* = 13.1 Hz, 4.0 Hz, 1H, H-18), 1.34 (s, 3H, CH_3), 1.30 (s, 3H, CH_3), 1.02 (s, 3H, CH_3), 0.99 (s, 3H, CH_3), 0.94 (s, 3H, CH_3), 0.93 (s, 3H, CH_3), 0.92 (s, 3H, CH_3); ^{13}C-NMR (150 MHz, pyridine-d_5) δ: 177.0, 145.0, 122.7, 107.4, 88.7, 76.8, 75.4, 73.2, 70.3, 62.4, 55.8, 51.8, 47.9, 46.8, 46.2, 42.3, 42.1, 39.8, 39.5, 38.8, 37.0, 34.4, 33.6, 33.5, 33.1 (C×2), 32.5, 30.8, 28.2, 27.9, 26.6, 26.0, 24.2, 24.1, 23.8, 23.7 (C×2), 18.5, 17.6, 17.0, 15.5. HRMS (ESI): Calcd for $[M + Na]^+$ $C_{41}H_{67}NNaO_7$: 708.4815, found 708.4805.

*28-N-normal-Butyl ursolic amide 3-O-β-D-glucopyranoside **4a***

White solid, 87% yield; $[\alpha]_D^{25}$: +17.25 (c 0.13, MeOH); ^{1}H-NMR (600 MHz, pyridine-d_5) δ: 77.14 (t, *J* = 5.7 Hz, 1H, N-H), 5.46 (t, *J* = 3.4 Hz, 1H, H-12), 4.98 (d,

J = 7.6 Hz, 1H, H-1′), 4.62 (dd, *J* = 11.6 Hz, 2.0 Hz, 1H, Glu-H), 4.43 (dd, *J* = 11.6 Hz, 5.3 Hz, 1H, Glu-H), 4.29−4.22 (m, 2H, Glu-H), 4.08−4.02 (m, 2H, Glu-H), 3.48−3.43 (m, 2H, H-3, H-31-1), 3.41−3.35 (m, 1H, H-31-2), 2.39 (d, *J* = 10.7 Hz, 1H, H-18), 1.35 (s, 3H, CH_3), 1.24 (s, 3H, CH_3), 1.04 (s, 3H, CH_3), 1.00 (s, 3H, CH_3), 0.97 (d, J = 6.4 Hz, 3H, CH_3), 0.95 (s, 3H, CH_3), 0.91 (s, 3H, CH_3), 0.86 (t, *J* = 7.3 Hz, 3H, H-34); ^{13}C-NMR (150 MHz, pyridine-d_5) δ: 177.2, 139.8, 125.7, 106.9, 88.9, 78.8, 78.2, 75.8, 72.0, 63.2, 55.9, 53.6, 47.9 (C×2), 42.6, 40.0, 39.9, 39.6, 39.5, 39.3, 38.9, 38.2, 36.9, 33.4, 32.1, 31.2, 28.4, 28.3, 26.6, 24.9, 23.8, 23.6, 21.3, 20.5, 18.5, 17.5 (C×2), 17.0, 15.6, 13.9; HRMS (ESI): Calcd for $[M + H]^+$ $C_{40}H_{68}NO_7$: 674.4996, found 674.4989.

28-N-Butoxypropyl ursolic amide 3-O-β-D-glucopyranoside ***4b***

White solid, 84% yield; $[\alpha]^{25}_{D}$: +23.25 (c 0.13, MeOH); ^{1}H-NMR (600 MHz, pyridine-d_5) δ: 7.26 (t, *J* = 5.4 Hz, 1H, N-H), 5.47 (t, *J* = 3.5 Hz, 1H, H-12), 4.97 (d, *J* = 7.7 Hz, 1H, H-1′), 4.62 (dd, *J* = 11.6 Hz, 2.1 Hz, 1H, Glu-H), 4.43 (dd, *J* = 11.6 Hz, 5.4 Hz, 1H, Glu-H), 4.29−4.24 (m, 2H, Glu-H), 4.08−4.02 (m, 2H, Glu-H), 3.66−3.61 (m, 1H, H-31-1), 3.56−3.52 (m, 1H, H-31-1), 3.51−3.47 (m, 2H, H-33), 3.44 (dd, *J* = 11.8 Hz, 4.5 Hz, 1H, H-3), 3.37 (t, *J* = 6.6 Hz, 1H, H-34), 2,39 (d, *J* = 10.6 Hz, 1H, H-18), 1.35 (s, 3H, CH_3), 1.24 (s, 3H, CH_3), 1.04 (s, 3H, CH_3), 1.00−0.99 (m, 6H, 2×CH_3), 0.96 (s, 3H, CH_3), 0.92 (s, 3H, CH_3), 0.87 (t, *J* = 7.4 Hz, 3H, H-37); ^{13}C-NMR (150 MHz, pyridine-d_5) δ: 177.2, 139.6, 125.7, 106.9, 88.9, 78.7, 78.2, 75.7, 71.9, 70.8, 69.5, 63.1, 55.8, 53.6, 47.9, 47.7, 42.5, 40.0, 39.8, 39.5, 39.4, 38.9, 38.1, 37.9, 36.9, 33.4, 32.2, 31.2, 30.1, 28.3, 28.2, 26.6, 24.8, 23.8, 23.6, 21.3, 19.6, 18.4, 17.5, 17.4, 17.0, 15.6, 14.0; HRMS (ESI): Calcd for $[M + Na]^+$ $C_{43}H_{73}NNaO_8$: 754.5234, found 754.5234.

28-N-(4’-Hydroxyphenylethyl) ursolic amide 3-O-β-D-glucopyranoside ***4c***

White solid, 61% yield; $[\alpha]^{25}_{D}$: +17.25 (c 0.13, MeOH); ^{1}H-NMR (600 MHz, pyridine-d_5) δ: 7.25−7.23 (m, 2H, Ph-H), 7.18−7.17 (m, 2H, Ph-H), 7.06 (t, *J* = 5.7 Hz,

1H, N-H), 5.31 (t, *J* = 3.2 Hz, 1H, H-12), 4.97 (d, *J* = 7.8 Hz, 1H, H-1′), 4.61 (dd, *J* = 11.6 Hz, 2.3 Hz, 1H, Glu-H), 4.43 (dd, *J* = 11.6 Hz, 5.6 Hz, 1H, Glu-H), 4.28−4.22 (m, 2H, Glu-H), 4.08−4.02 (m, 2H, Glu-H), 3.90−3.85 (m, 1H, H-31-1), 3.57−3.52 (m, 1H, H-31-2), 3.43 (dd, *J* = 11.9 Hz, 4.5 Hz, 1H, H-3), 2.98−2.94 (m, 2H, H-32-1, H-18), 2.88−2.83 (m, 1H, H-32-2), 1.35 (s, 3H, CH_3), 1.21 (s, 3H, CH_3), 1.03 (s, 3H, CH_3), 0.96−0.93 (m, 6H, 2×CH_3), 0.92−0.90 (m, 6H, 2×CH_3); ^{13}C-NMR (150 MHz, pyridine-d_5) δ: 177.4, 157.5, 139.5, 130.3 (C×2), 126.0, 116.4, 106.9, 88.9, 78.7, 78.3, 75.8, 71.9, 63.1, 55.8, 47.9, 47.8, 42.5, 41.7, 40.0, 39.9, 39.5, 39.3, 38.8, 38.1, 36.8, 35.3, 33.2, 31.2, 28.3 (C×2), 26.6, 25.0, 23.7, 23.6, 21.3, 18.4, 17.5, 17.0, 15.6; HRMS (ESI): Calcd for $[M + Na]^+$ $C_{44}H_{67}NNaO_8$: 760.4764, found 760.4761.

28-N-Cyclopentyl ursolic amide 3-O-β-D-glucopyranoside ***4d***

White solid, 85% yield; $[\alpha]^{25}_{D}$: +24.00 (c 0.13, MeOH); ^{1}H-NMR (600 MHz, pyridine-d_5) δ: 6.70 (d, *J* = 6.6 Hz, 1H, N-H), 5.48 (t, *J* = 3.5 Hz, 1H, H-12), 4.97 (d, *J* = 7.8 Hz, 1H, H-1′), 4.62 (dd, *J* = 11.6 Hz, 2.3 Hz, 1H, Glu-H), 4.46−4.42 (m, 2H, H-31, Glu-H), 4.29−4.22 (m, 2H, Glu-H), 4.08−4.02 (m, 2H, Glu-H), 3.44 (dd, *J* = 11.7 Hz, 4.4 Hz, 1H, H-3), 2.34 (d, *J* = 10.8 Hz, 1H, H-18), 1.36 (s, 3H, CH_3), 1.23 (s, 3H, CH_3), 1.05 (s, 3H, CH_3), 1.01 (s, 3H, CH_3), 0.95 (d, *J* = 6.4 Hz, 3H, CH_3), 0.93 (s, 3H, CH_3), 0.92 (s, 3H, CH_3); ^{13}C-NMR (150 MHz, pyridine-d_5) δ: 177.0, 139.8, 125.6, 106.9, 88.9, 78.7, 78.2, 75.8, 71.9, 63.1, 55.8, 53.6, 51.5, 47.9, 47.6, 42.7, 40.0, 39.9, 39.5, 39.2, 38.9, 38.0, 36.9, 33.4 (C×2), 32.6, 31.2, 28.3 (C×2), 26.6, 24.8, 24.1 (C×2), 23.7, 23.6, 21.3, 18.4, 17.6, 17.4, 17.0, 15.6; HRMS (ESI): Calcd for $[M + Na]^+$ $C_{41}H_{67}NNaO_7$: 708.4815, found 708.4811

28-N-normal-Butyl ursolic amide 3-O-β-D-galactopyranoside ***5a***

White solid, 83% yield; $[\alpha]^{25}_{D}$: +18.00 (c 0.13, MeOH); ^{1}H-NMR (600 MHz, pyridine-d_5) δ: 7.14 (t, *J* = 5.7 Hz, 1H, N-H), 5.47 (t, *J* = 3.4 Hz, 1H, H-12), 4.89 (d, *J* = 7.8 Hz, 1H, H-1′), 4.60−4.59 (m, 1H, Gal-H), 4.52−4.46 (3H, m, Gal-H), 4.19 (dd, *J* = 3.3 Hz, 9.5 Hz, 1H, Gal-H), 4.14 (t, *J* = 6.0 Hz, 1H, Gal-H), 3.48−3.42 (m, 2H, H-3,

H-31-1), 3.41−3.35 (m, 1H, H-31-2), 2.40 (d, *J* = 10.8 Hz, 1H, H-18), 1.34 (s, 3H, CH_3), 1.25 (s, 3H, CH_3), 1.00 (s, 3H, CH_3), 0.99 (s, 3H, CH_3), 0.98 (d, *J* = 6.5 Hz, 3H, CH_3), 0.95 (s, 3H, CH_3), 0.92 (s, 3H, CH_3), 0.86 (t, *J* = 7.3 Hz, 3H, H-34); ^{13}C-NMR (150 MHz, pyridine-d_5) δ: 177.2, 139.8, 125.7, 107.5, 88.8, 76.8, 75.4, 73.2, 70.3, 62.5, 55.9, 53.6, 47.9, 47.8, 42.6, 40.0, 39.9, 39.5 (C ×2), 39.3, 38.9, 38.2, 36.9, 33.4, 32.1, 31.2, 28.3 (C ×2), 26.7, 24.9, 23.8, 23.6, 21.3, 20.5, 18.4, 17.5 (C ×2), 17.0, 15.6, 13.9; HRMS (ESI): Calcd for $[M + Na]^+$ $C_{40}H_{67}NNaO_7$: 696.4815, found 696.4807.

28-N-Butoxypropyl ursolic amide 3-O-β-D-galactopyranoside ***5b***

White solid, 86% yield; $[\alpha]^{25}_{D}$: +24.00 (c 0.13, MeOH); ^{1}H-NMR (600 MHz, pyridine-d_5) δ: 7.26 (t, *J* = 5.3 Hz, 1H, N-H), 5.47 (t, *J* = 3.4 Hz, 1H, H-12), 4.89 (d, *J* = 7.7 Hz, 1H, H-1′), 4.60−4.59 (m, 1H, Gal-H), 4.52−4.46 (m, 3H, Gal-H), 4.19 (dd, *J* = 3.4 Hz, 9.4 Hz, 1H, Gal-H), 4.14 (t, *J* = 6.0 Hz, 1H, Gal-H), 3.66−3.61 (m, 1H, H-31-1), 3.56−3.52 (m, 1H, H-31-1), 3.51−3.47 (m, 2H, H-33), 3.44 (dd, *J* = 11.7 Hz, 4.2 Hz, 1H, H-3), 3.37 (t, *J* = 6.5 Hz, 1H, H-34), 2,39 (d, *J* = 10.7 Hz, 1H, H-18), 1.34 (s, 3H, CH_3), 1.25 (s, 3H, CH_3), 1.01−0.99 (m, 9H, 3×CH_3), 0.96 (s, 3H, CH_3), 0.93 (s, 3H, CH_3), 0.87 (t, *J* = 7.4 Hz, 3H, H-37); ^{13}C-NMR (150 MHz, pyridine-d_5) δ: 177.2, 139.6, 125.7, 107.5, 88.8, 76.8, 75.4, 73.1, 70.8, 70.2, 69.5, 62.4, 55.8, 53.6, 47.9, 47.7, 42.5, 40.0, 39.8, 39.5, 39.4, 38.9, 38.1, 37.9, 36.9, 33.4, 32.2, 31.2, 30.1, 28.3, 28.2, 26.6, 24.8, 23.8, 23.6, 21.3, 19.6, 18.4, 17.5, 17.4, 17.0, 15.6, 14.0; HRMS (ESI): Calcd for $[M + H]^+$ $C_{43}H_{74}NO_8$: 732.5414, found 732.5405.

28-N-(4'-Hydroxyphenylethyl) ursolic amide 3-O-β-D-galactopyranoside ***5c***

White solid, 67% yield; $[\alpha]^{25}_{D}$: +15.75 (c 0.13, MeOH); ^{1}H-NMR (600 MHz, pyridine-d_5) δ: 7.25−7.24 (m, 2H, Ph-H), 7.19−7.17 (m, 2H, Ph-H), 7.08 (t, *J* = 5.7 Hz, 1H, N-H), 5.32 (t, *J* = 3.2 Hz, 1H, H-12), 4.89 (d, *J* = 7.7 Hz, 1H, H-1′), 4.61−4.60 (m, 1H, Gal-H), 4.53−4.47 (3H, m, Gal-H), 4.19 (dd, J = 3.4 Hz, 9.5 Hz, 1H, Gal-H), 4.14 (t, *J* = 6.1 Hz, 1H, Gal-H), 3.91−3.85 (m, 1H, H-31-1), 3.58−3.52 (m, 1H, H-31-2), 3.43 (dd, *J* = 11.9 Hz, 4.5 Hz, 1H, H-3), 2.99−2.94 (m, 2H, H-32-1, H-18), 2.88−2.84

(m, 1H, H-32-2), 1.34 (s, 3H, CH_3), 1.22 (s, 3H, CH_3), 1.00 (s, 3H, CH_3), 0.96−0.94 (m, 6H, 2×CH_3), 0.92−0.91 (m, 6H, 2×CH_3); ^{13}C-NMR (150 MHz, pyridine-d_5) δ: 177.4, 157.5, 139.5, 130.3 (C×2), 126.0, 116.3, 107.5, 88.8, 76.8, 75.4, 73.2, 70.3, 62.5, 55.8, 53.6, 47.9, 47.8, 42.5, 41.7, 40.0, 39.9, 39.5, 39.3, 38.9, 38.0, 36.8, 35.3, 33.2, 31.2, 28.3, 28.2, 26.7, 25.0, 23.7, 23.6, 21.3, 18.4, 17.5, 17.4, 17.0, 15.6; HRMS (ESI): Calcd for $[M + Na]^+$ $C_{44}H_{67}NNaO_8$: 760.4764, found 760.4761.

28-N-Cyclopentyl ursolic amide 3-O-β-D-galactopyranoside ***5d***

White solid, 82% yield; $[\alpha]^{25}_{D}$: +24.00 (c 0.13, MeOH); ^{1}H-NMR (600 MHz, pyridine-d_5) δ: 6.69 (d, J = 6.7 Hz, 1H, N-H), 5.48 (t, J = 3.4 Hz, 1H, H-12), 4.89 (d, J = 7.8 Hz, 1H, H-1′), 4.60−4.59 (m, 1H, Gal-H), 4.52−4.42 (m, 4H, H-31, Gal-H), 4.18 (dd, J = 3.5 Hz, 9.6 Hz, 1H, Gal-H), 4.14 (t, J = 6.1 Hz, 1H, Gal-H), 3.43 (dd, J = 11.7 Hz, 4.3 Hz, 1H, H-3), 2.34 (d, J = 10.8 Hz, 1H, H-18), 1.35 (s, 3H, CH_3), 1.24 (s, 3H, CH_3), 1.02 (s, 3H, CH_3), 1.01 (s, 3H, CH_3), 0.95 (d, J = 6.6 Hz, 3H, CH_3), 0.94 (s, 3H, CH_3), 0.93 (s, 3H, CH_3); ^{13}C-NMR (150 MHz, pyridine-d_5) δ: 176.9, 139.8, 125.7, 107.5, 88.8, 76.8, 75.4, 73.2, 70.3, 62.5, 55.9, 53.6, 51.5, 47.9, 47.6, 42.7, 40.0, 39.9, 39.5, 39.2, 38.9, 38.0, 36.9, 33.5, 33.4, 32.6, 31.2, 28.3 (C×2), 26.7, 24.8, 24.1 (C×2), 23.7, 23.6, 21.3, 18.4, 17.6, 17.4, 17.0, 15.7; HRMS (ESI): Calcd for $[M + Na]^+$ $C_{41}H_{67}NNaO_7$: 708.4815, found 708.4811.

The spectrogram of the compounds 2a−d, 3a−d, 4a−d, 5a−d

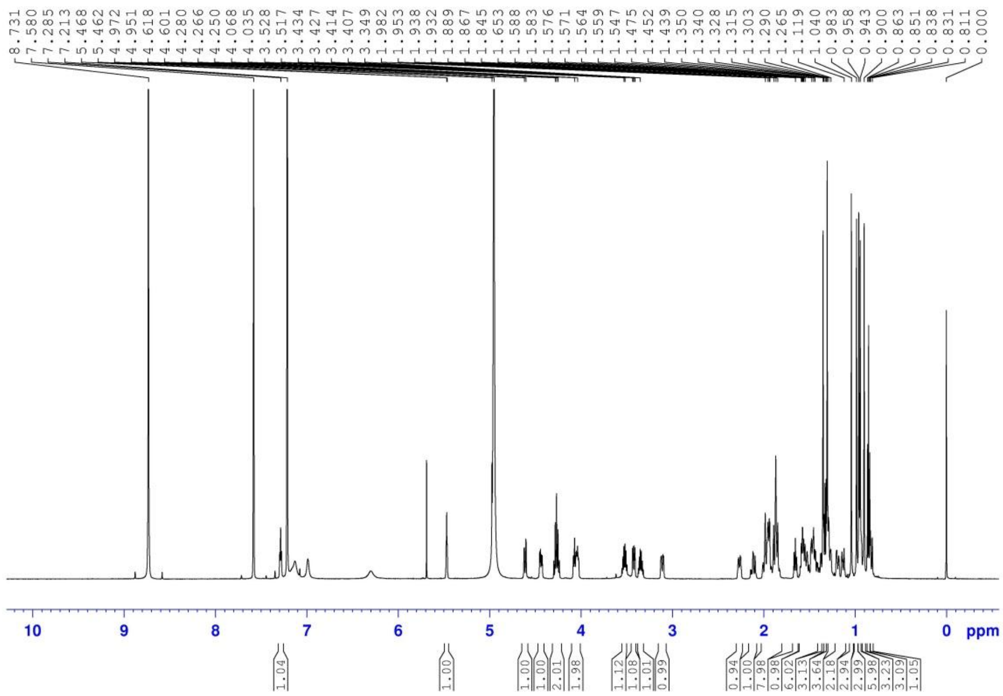

^{1}H NMR of compound **2a**

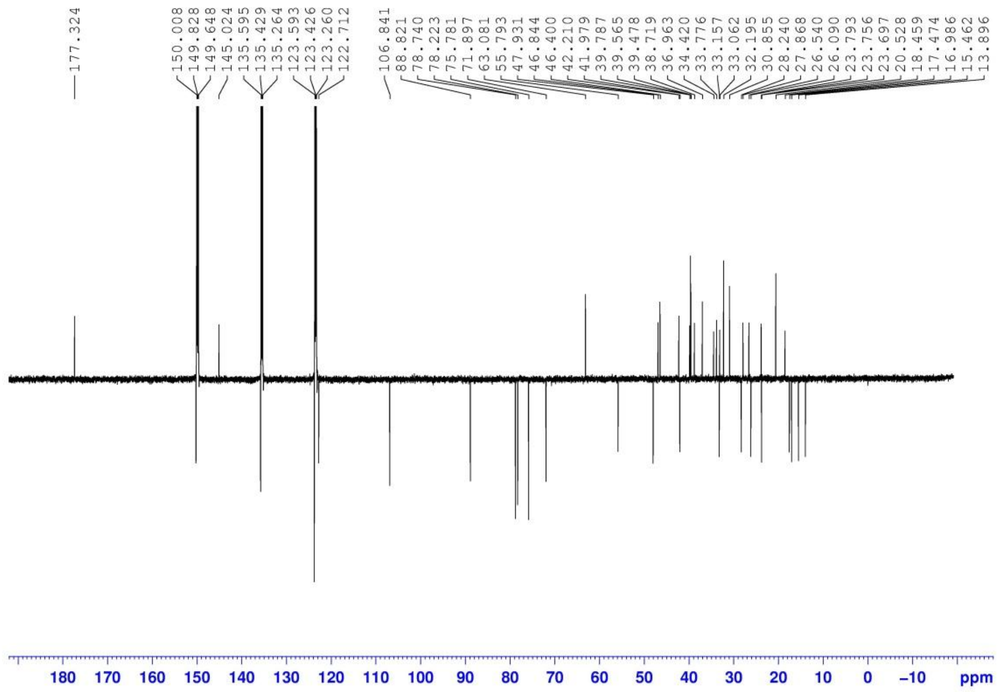

^{13}C NMR of compound **2a**

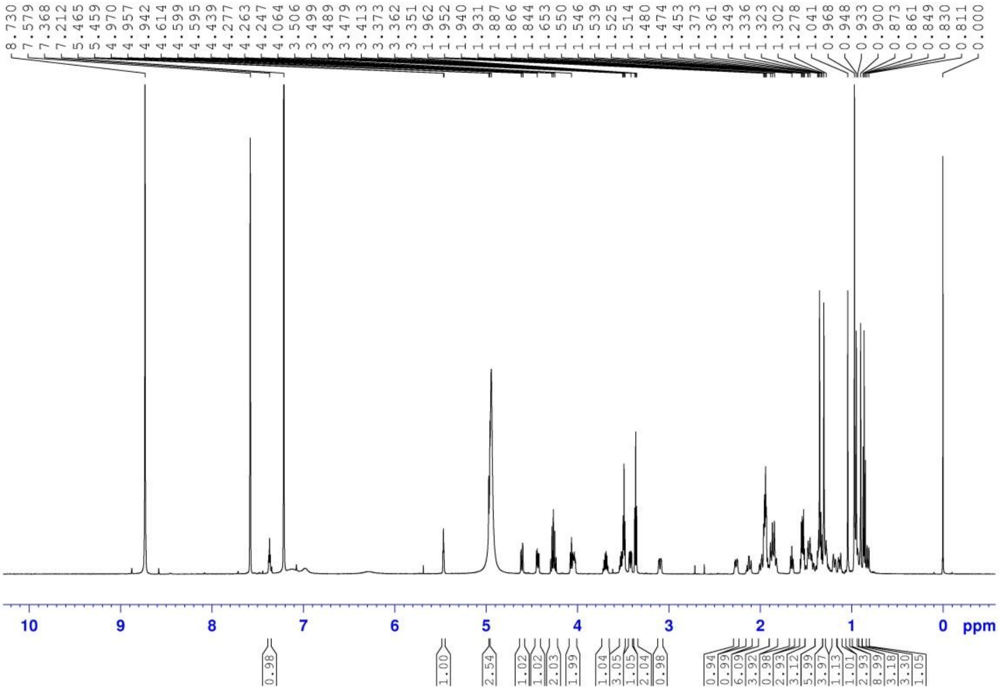

^{1}H NMR of compound **2b**

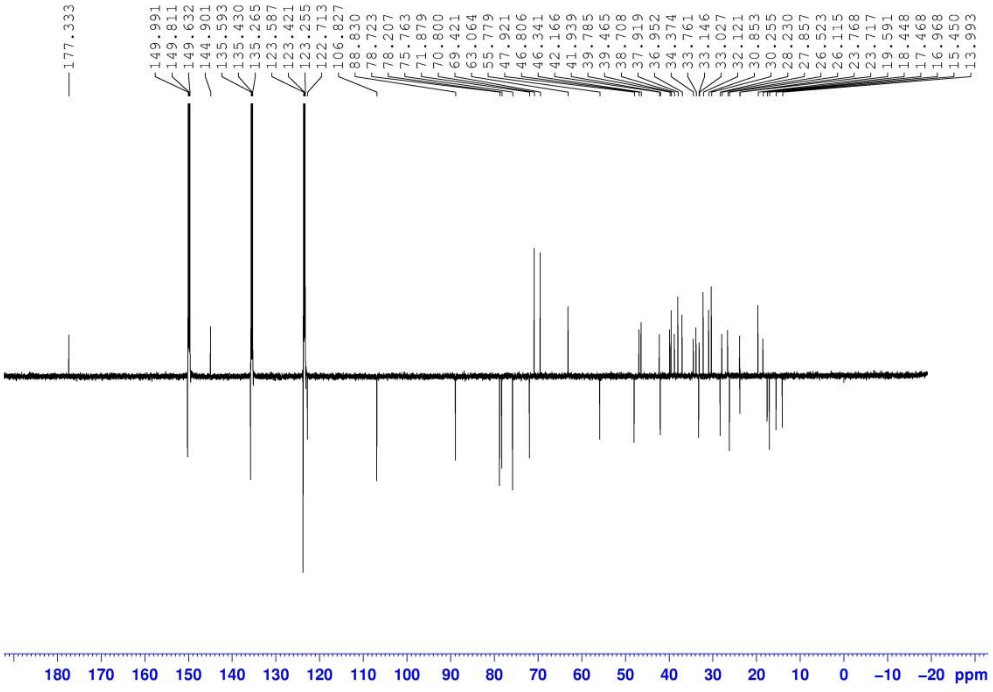

^{13}C NMR of compound **2b**

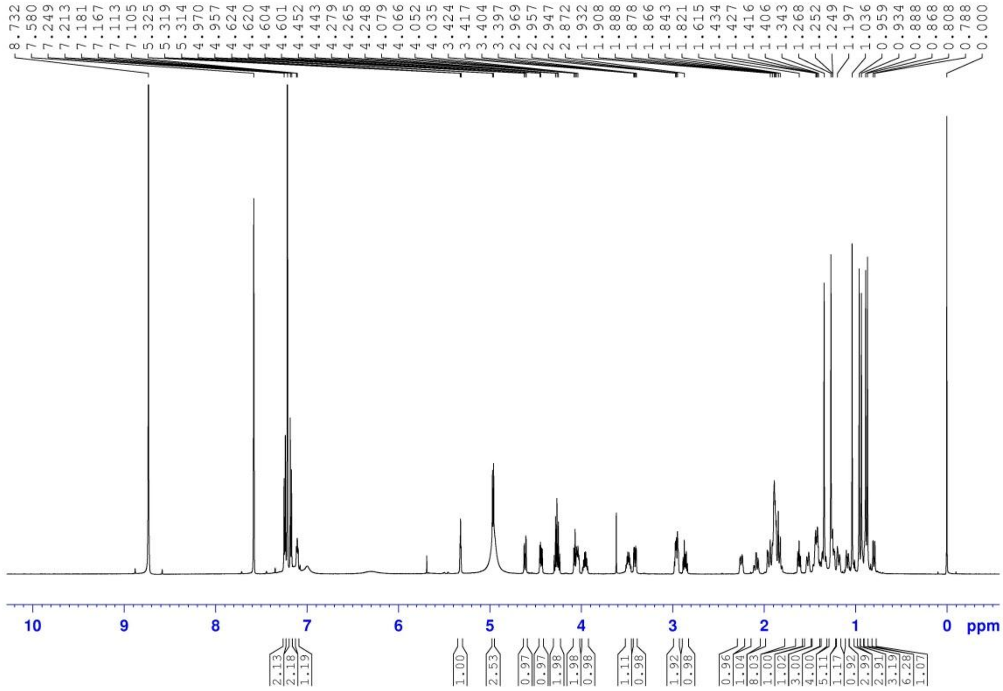

^{1}H NMR of compound **2c**

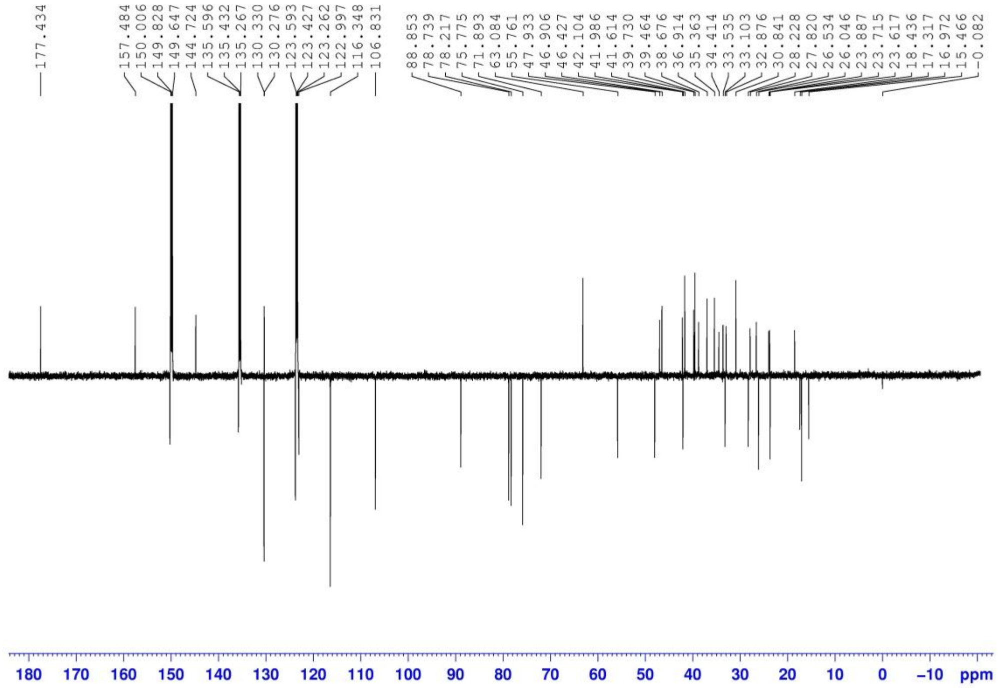

^{13}C NMR of compound **2c**

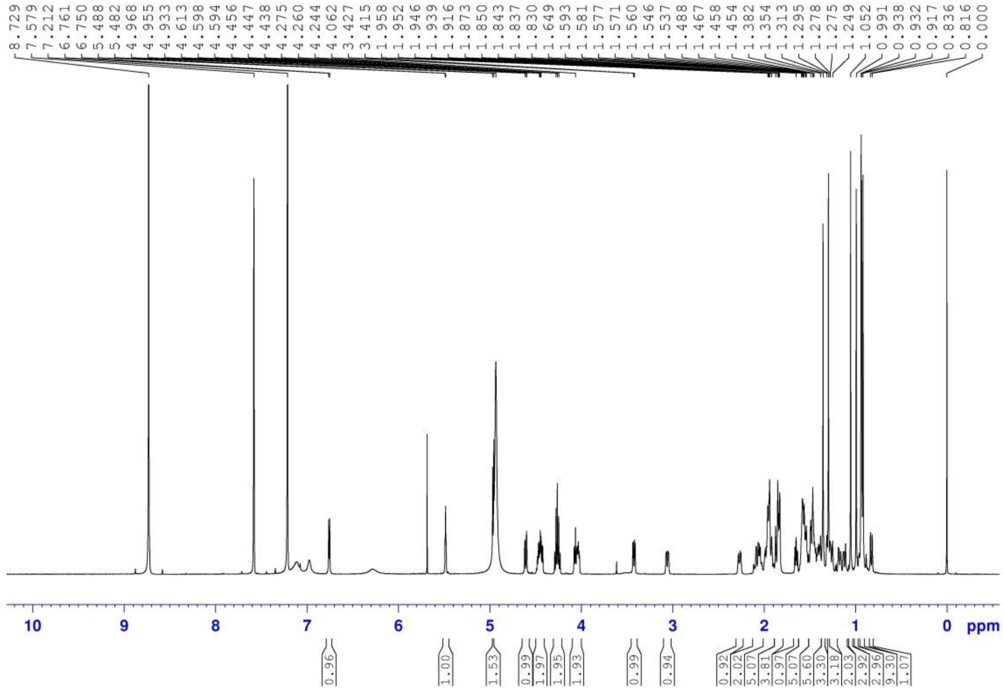

^{1}H NMR of compound **2d**

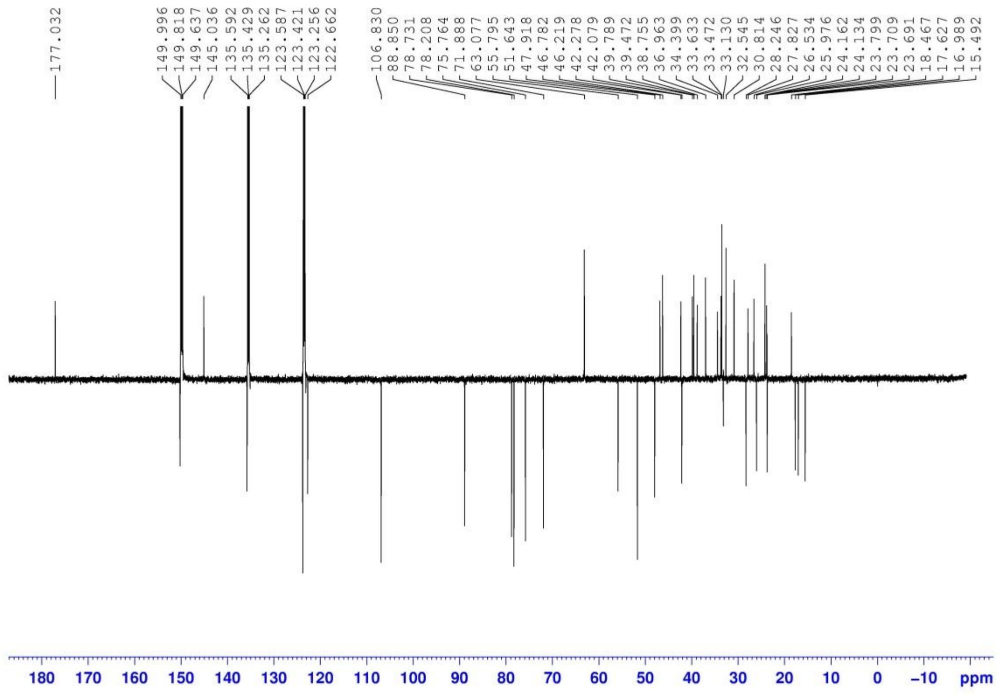

^{13}C NMR of compound **2d**

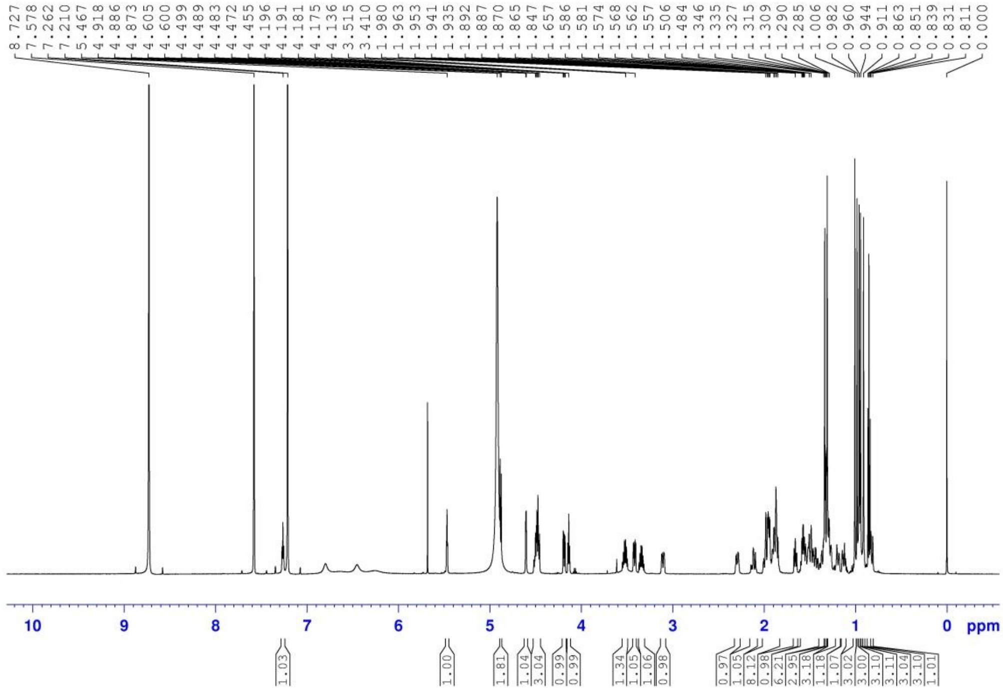

^{1}H NMR of compound **3a**

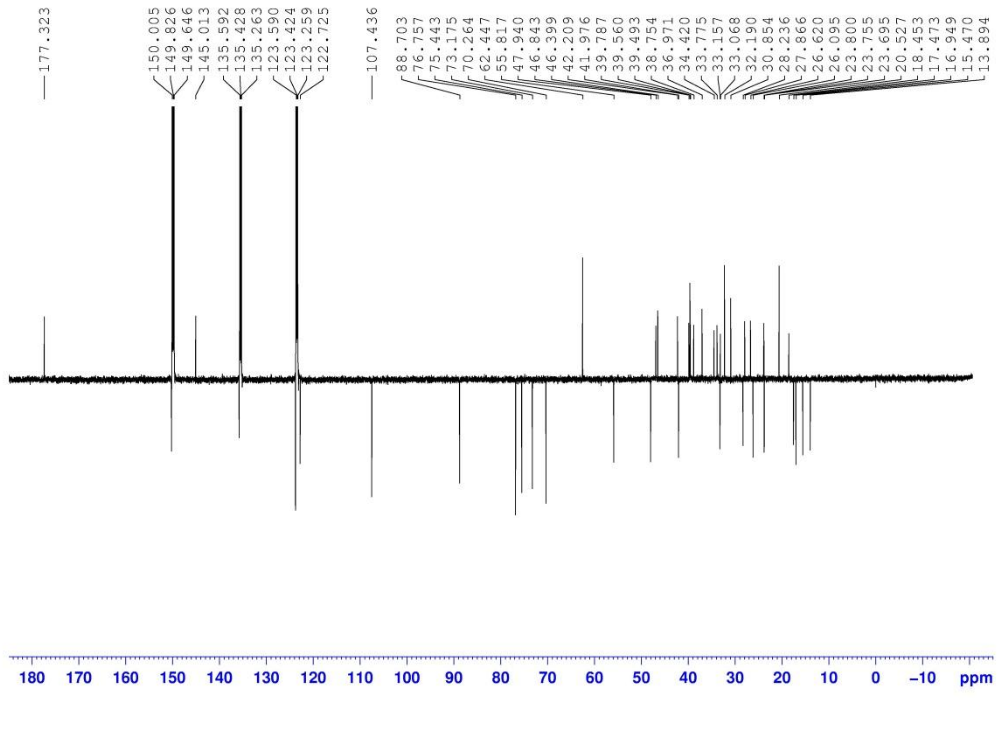

^{13}C NMR of compound **3a**

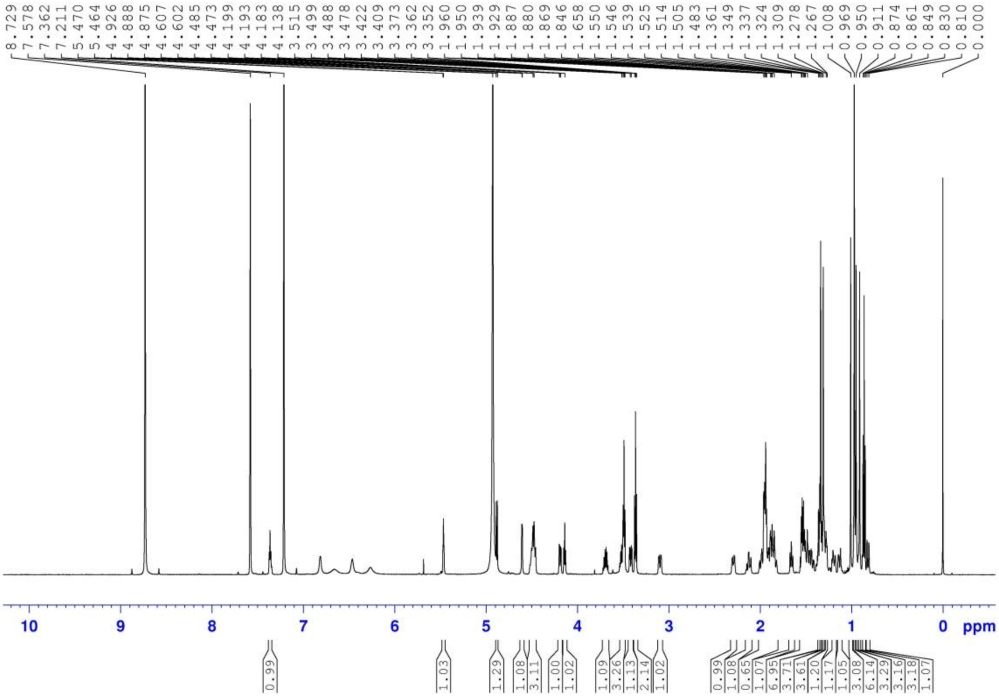

^{1}H NMR of compound **3b**

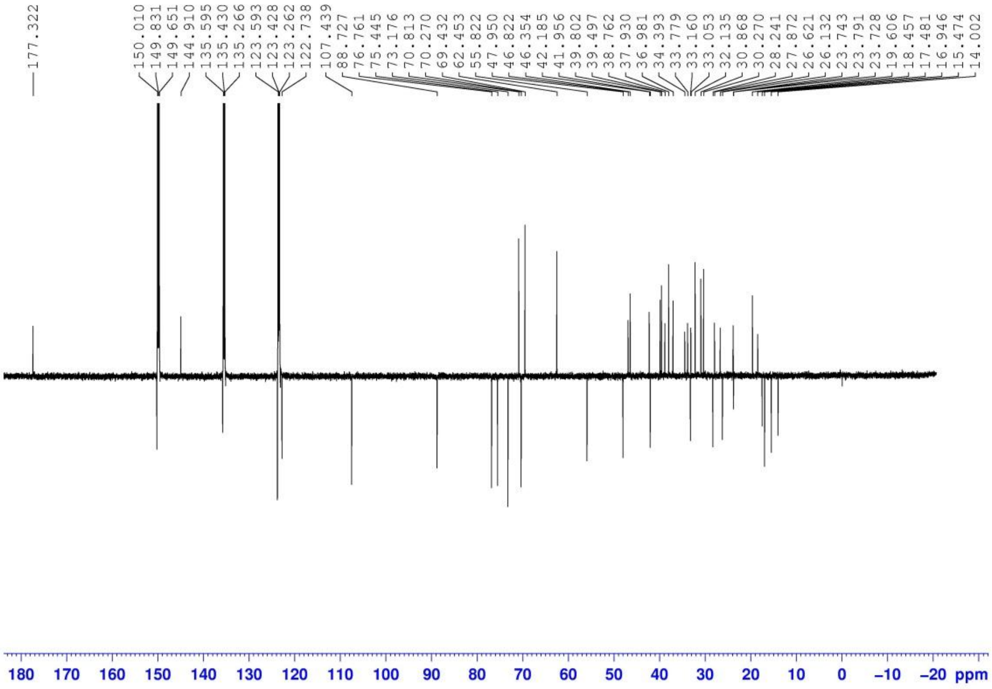

^{13}C NMR of compound **3b**

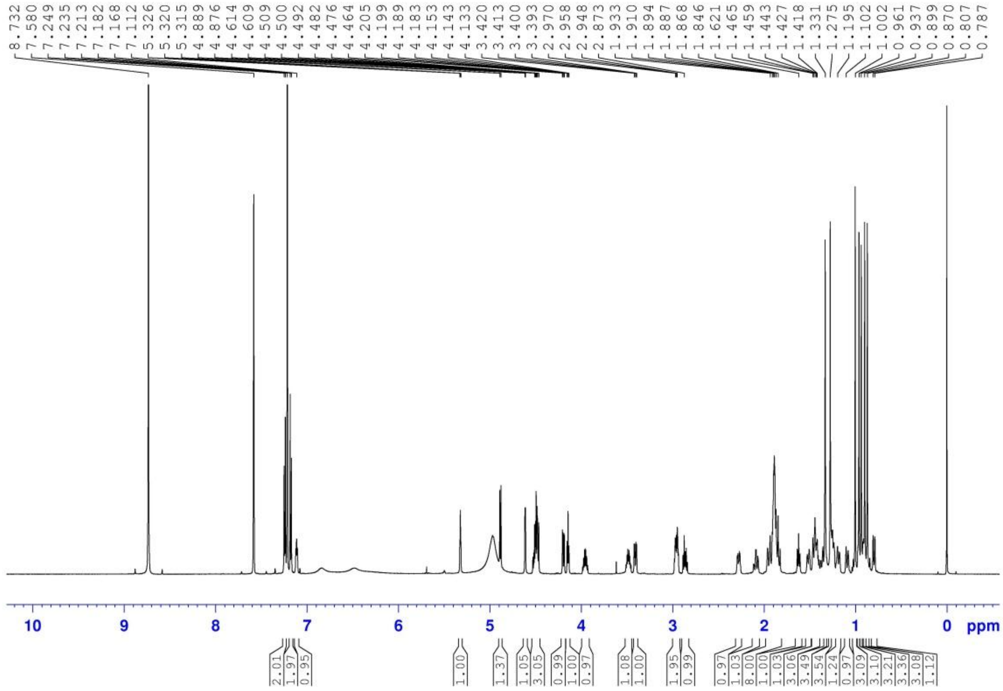

^{1}H NMR of compound **3c**

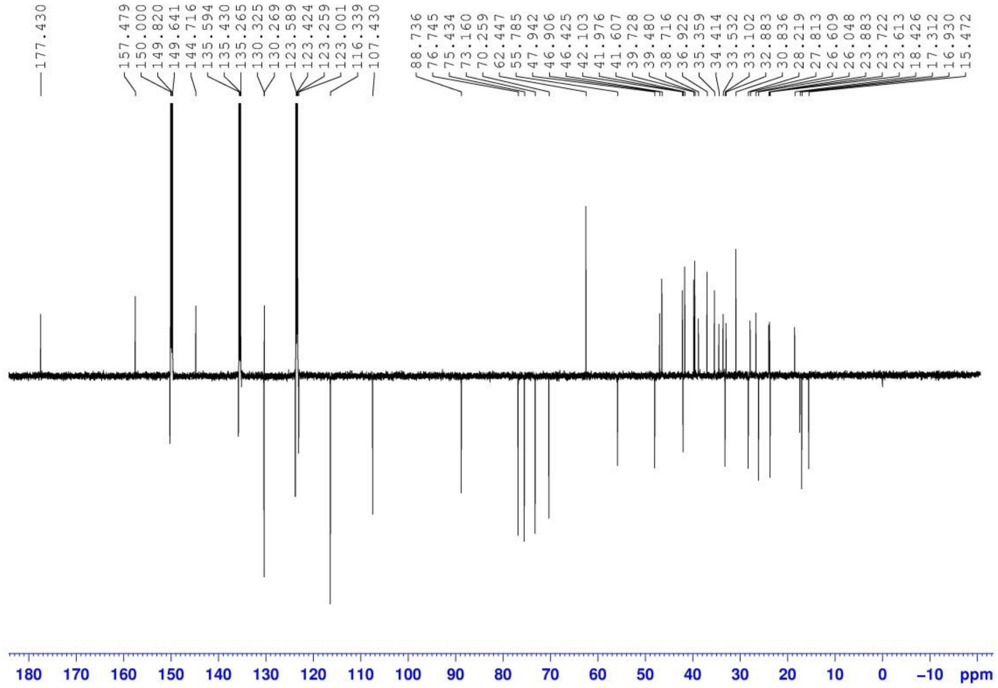

^{13}C NMR of compound **3c**

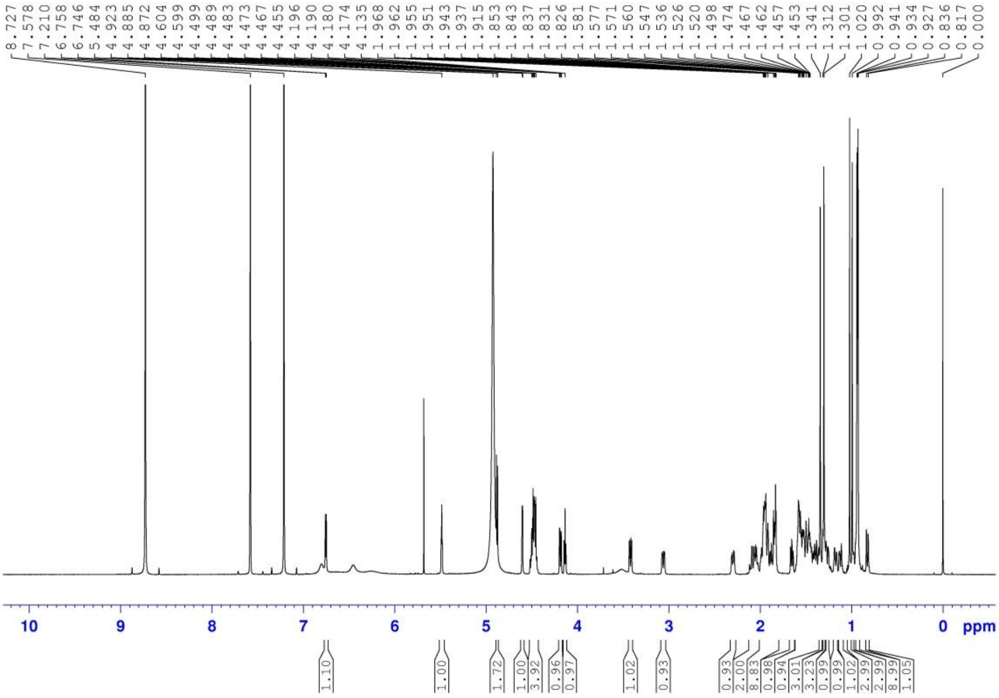

^{1}H NMR of compound **3d**

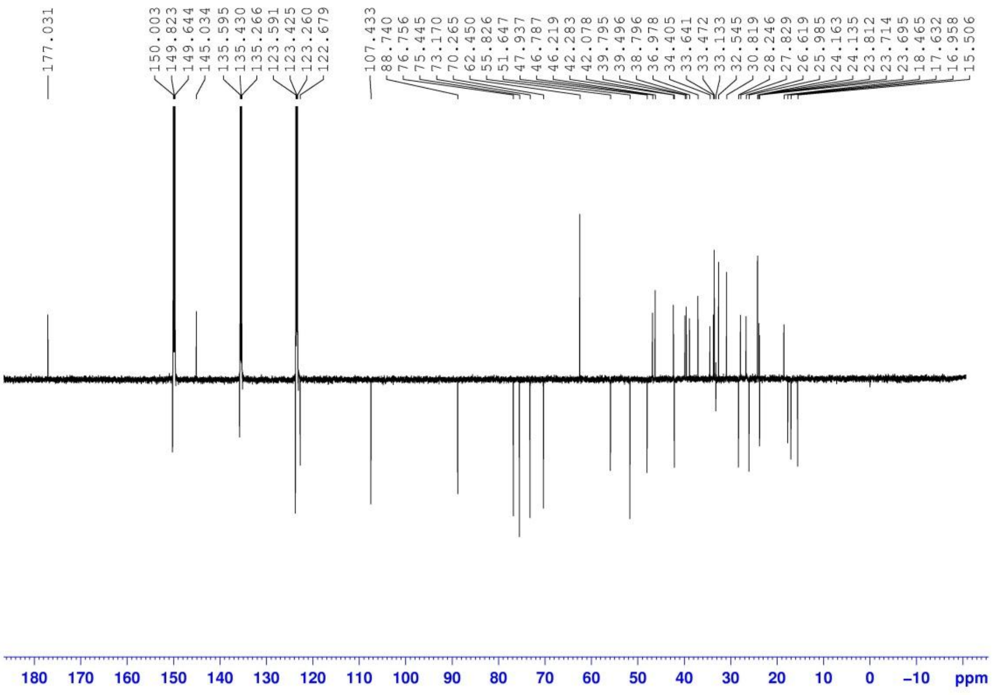

^{13}C NMR of compound **3d**

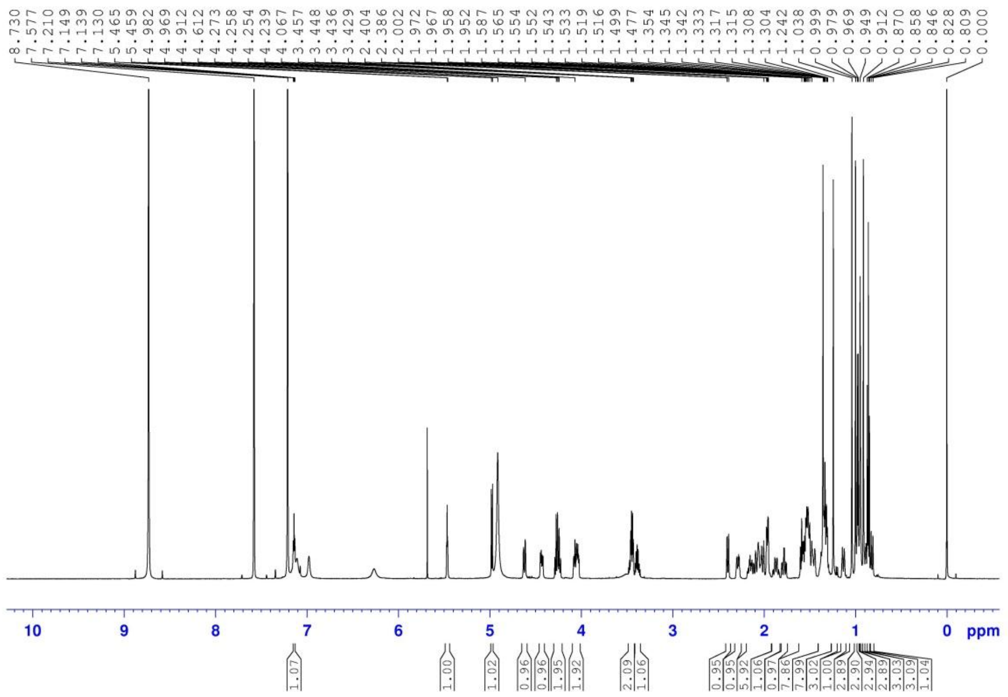

^{1}H NMR of compound **4a**

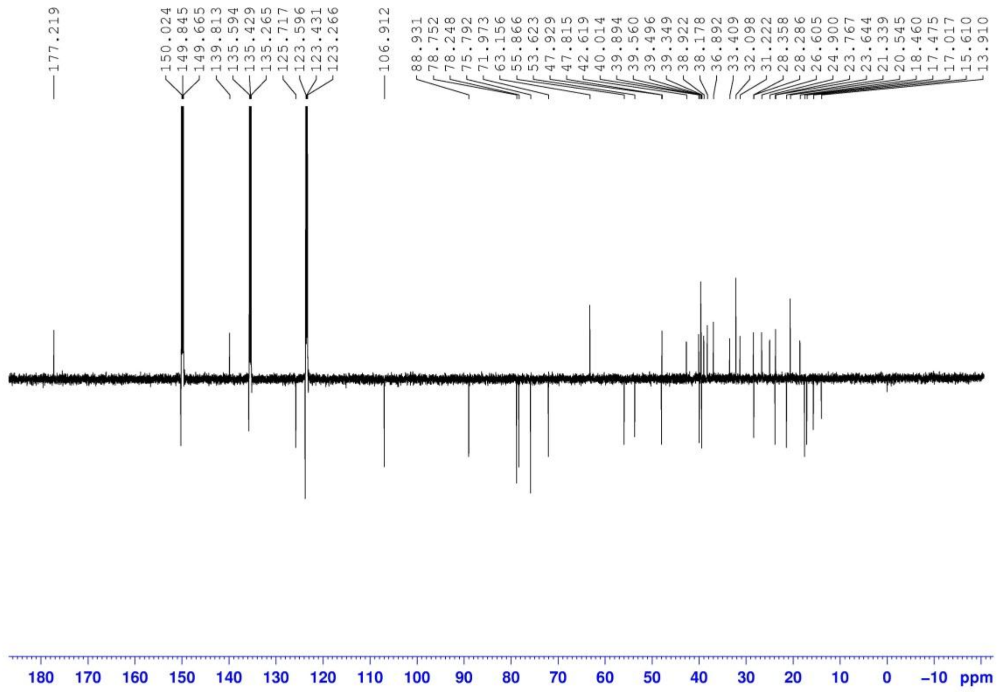

^{13}C NMR of compound **4a**

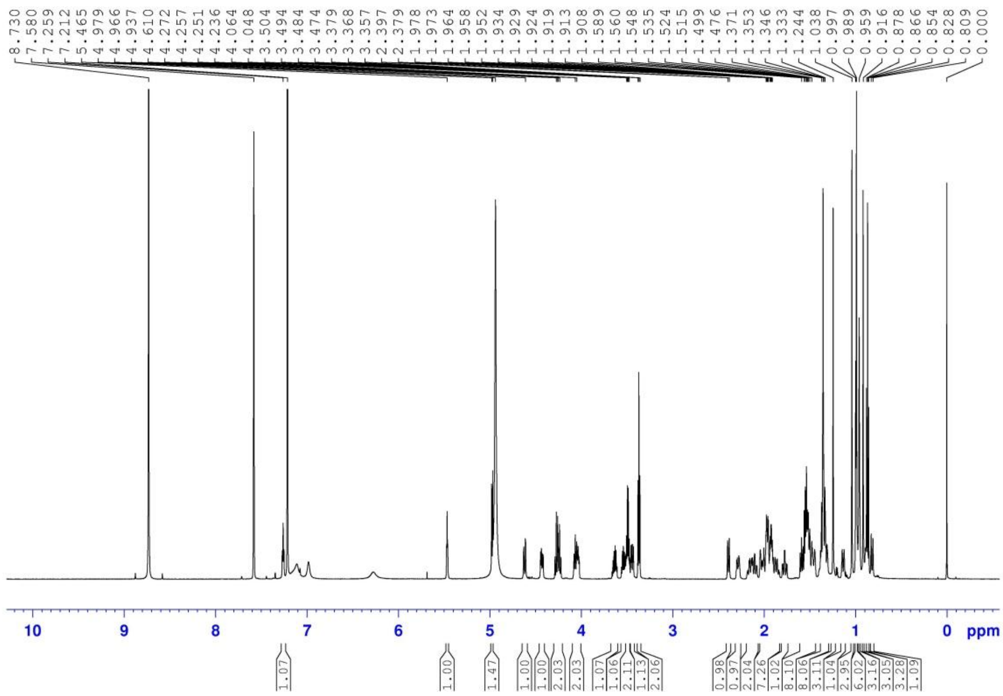

^{1}H NMR of compound **4b**

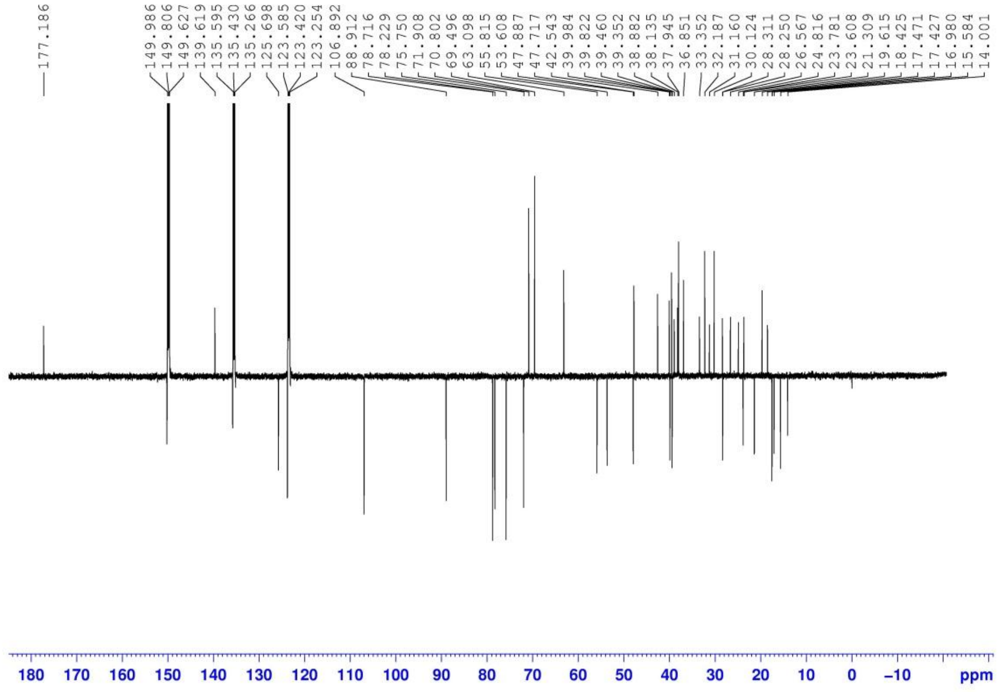

^{13}C NMR of compound **4b**

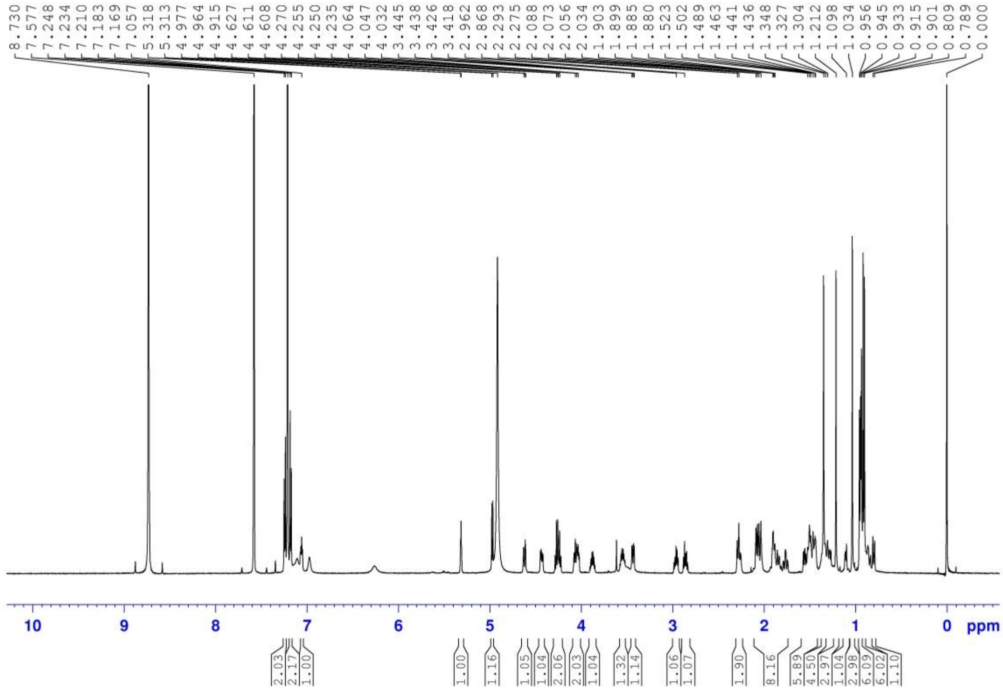

^{1}H NMR of compound **4c**

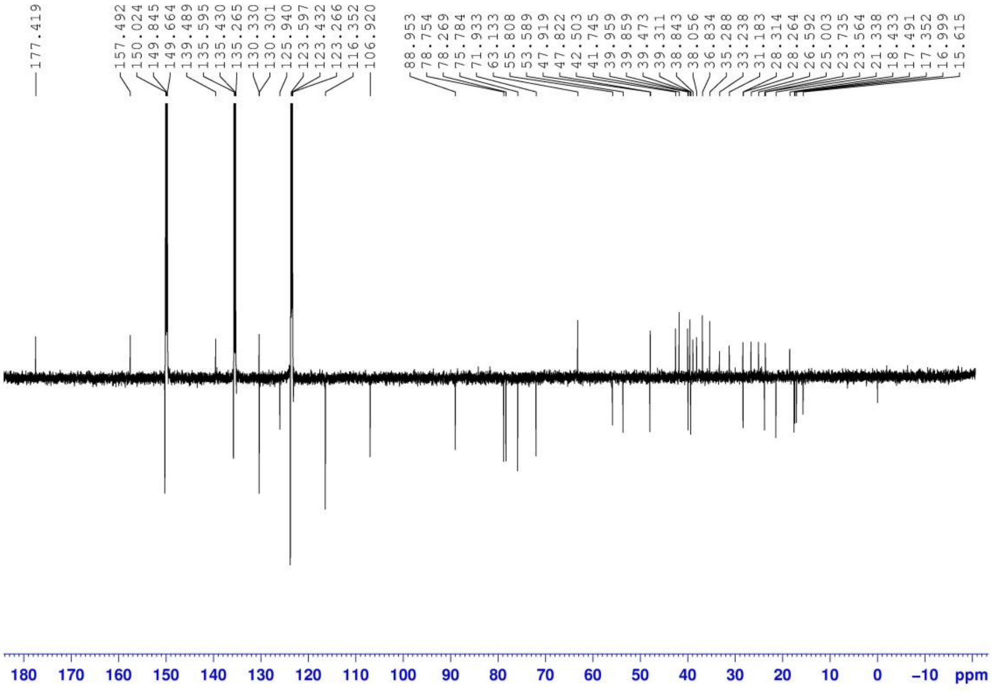

^{13}C NMR of compound **4c**

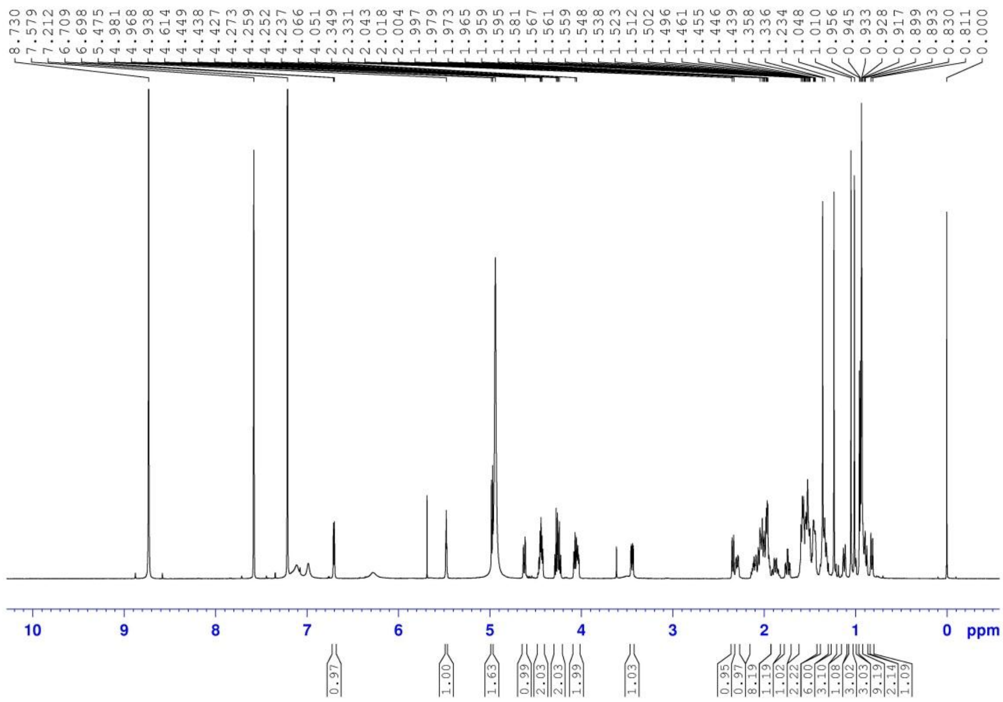

^{1}H NMR of compound **4d**

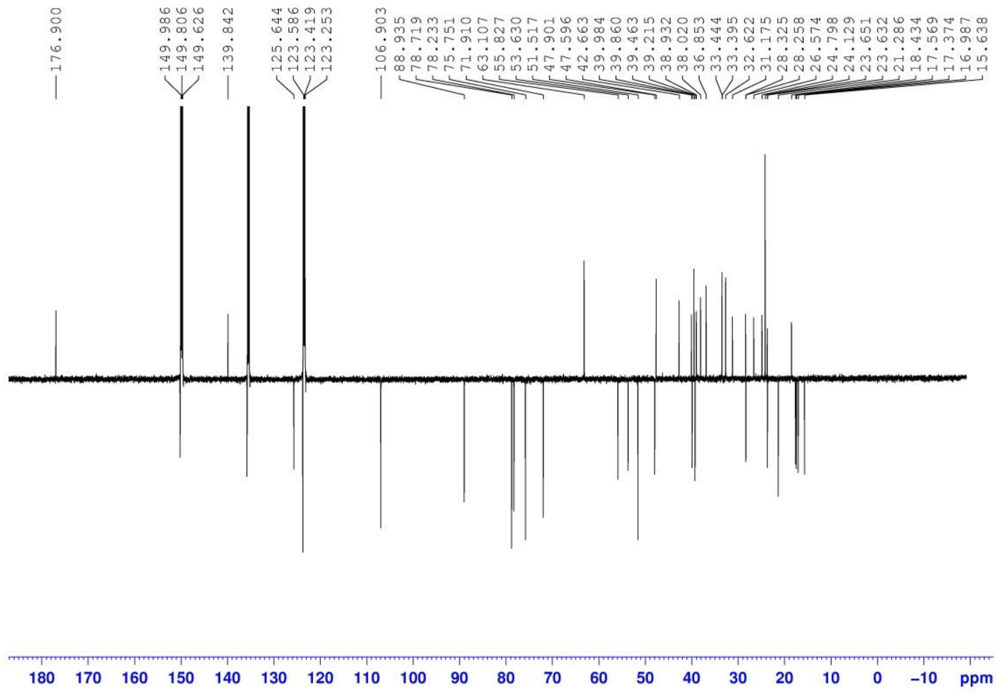

^{13}C NMR of compound **4d**

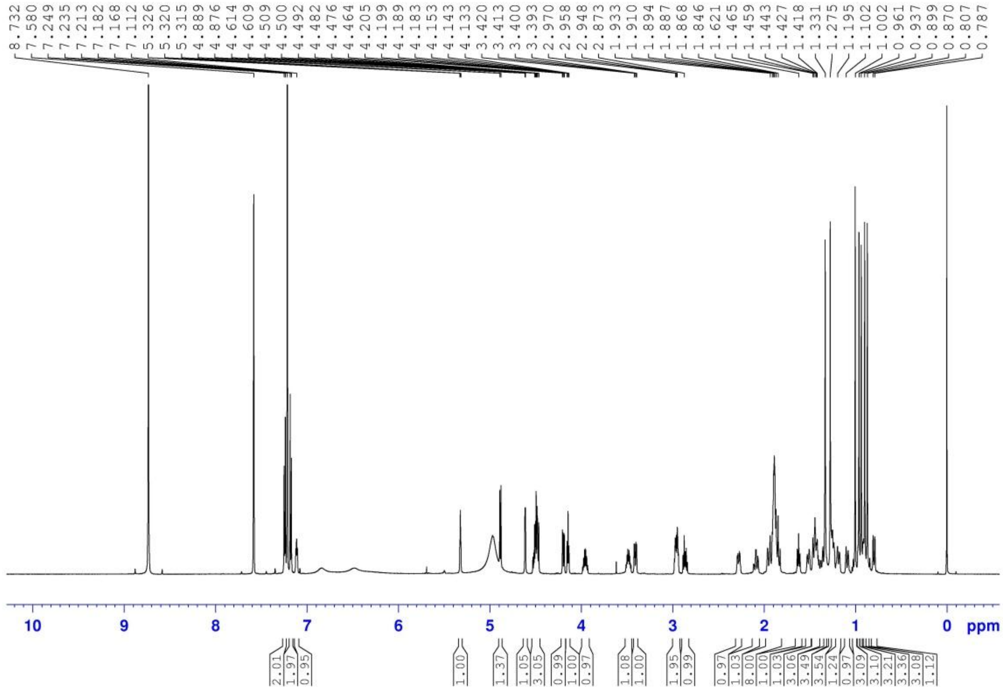

^{1}H NMR of compound **5a**

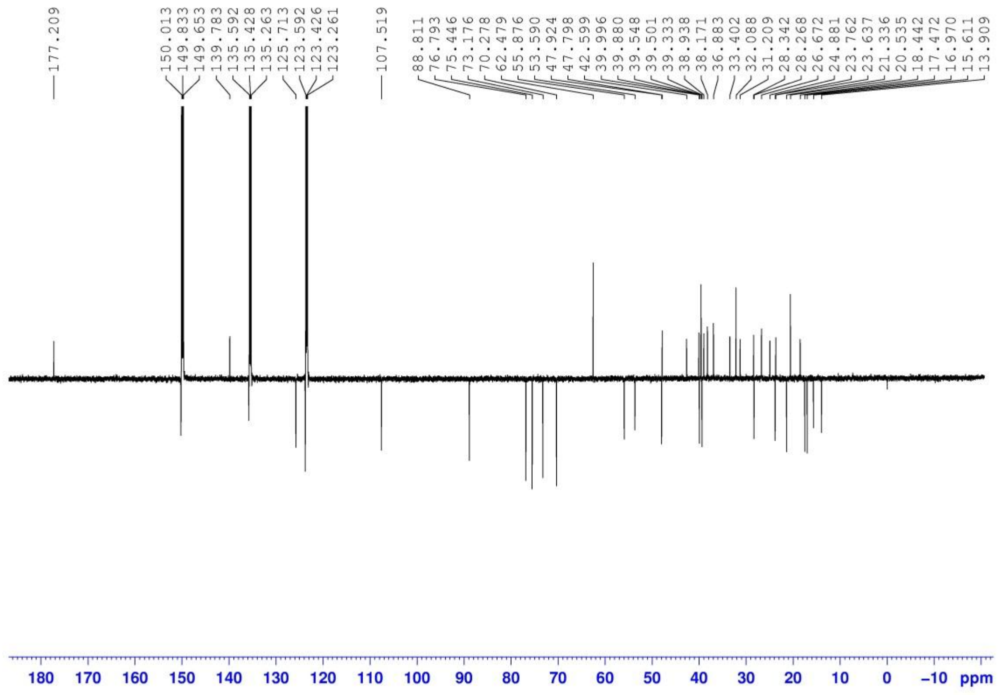

^{13}C NMR of compound **5a**

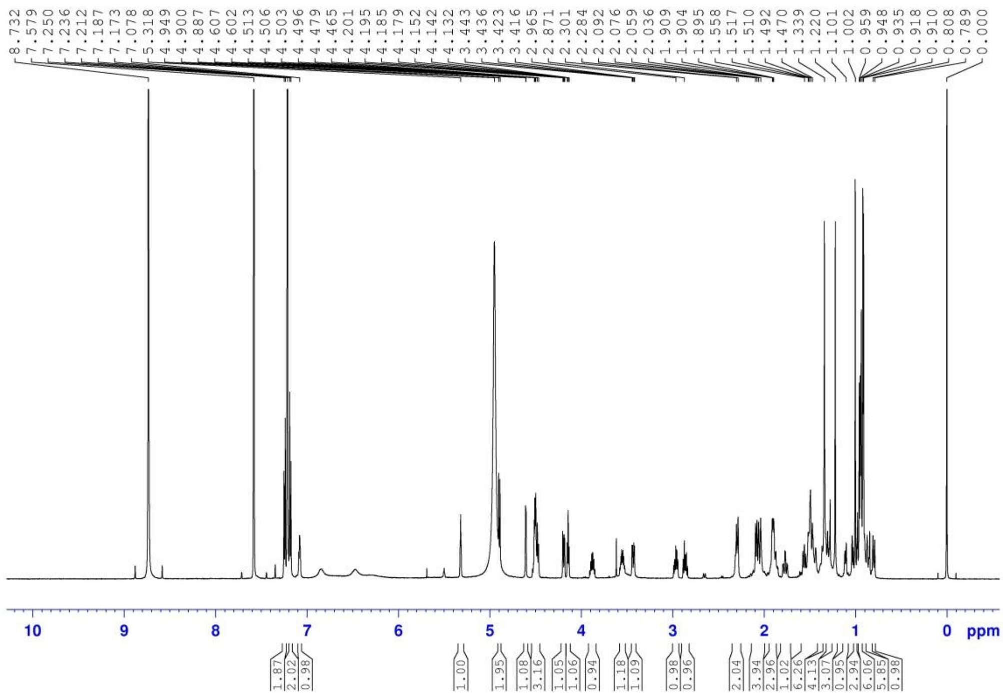

^{1}H NMR of compound **5b**

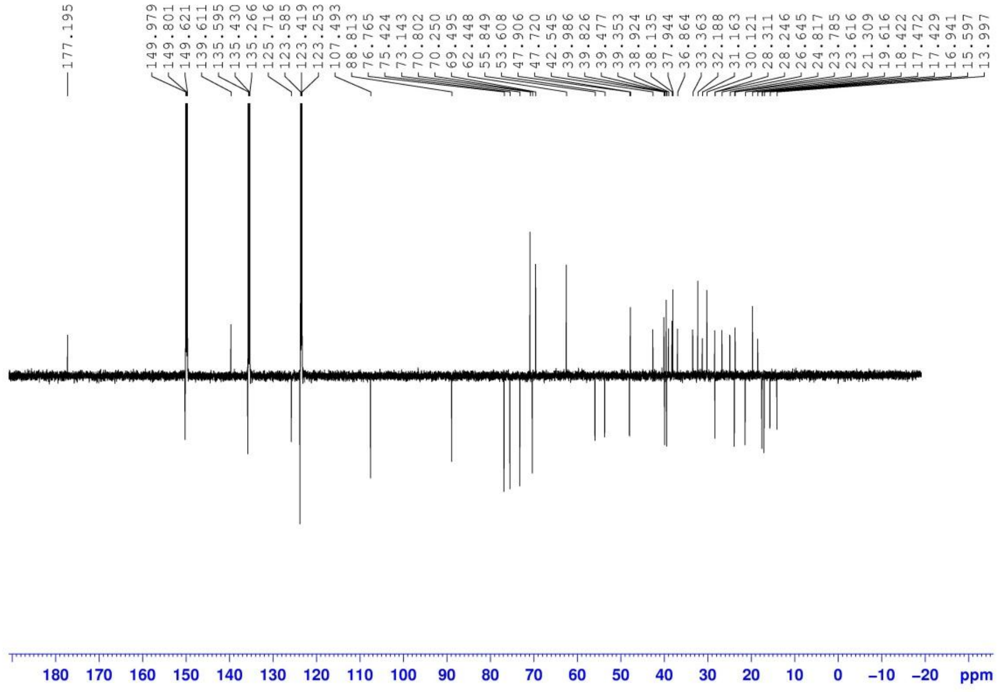

^{13}C NMR of compound **5b**

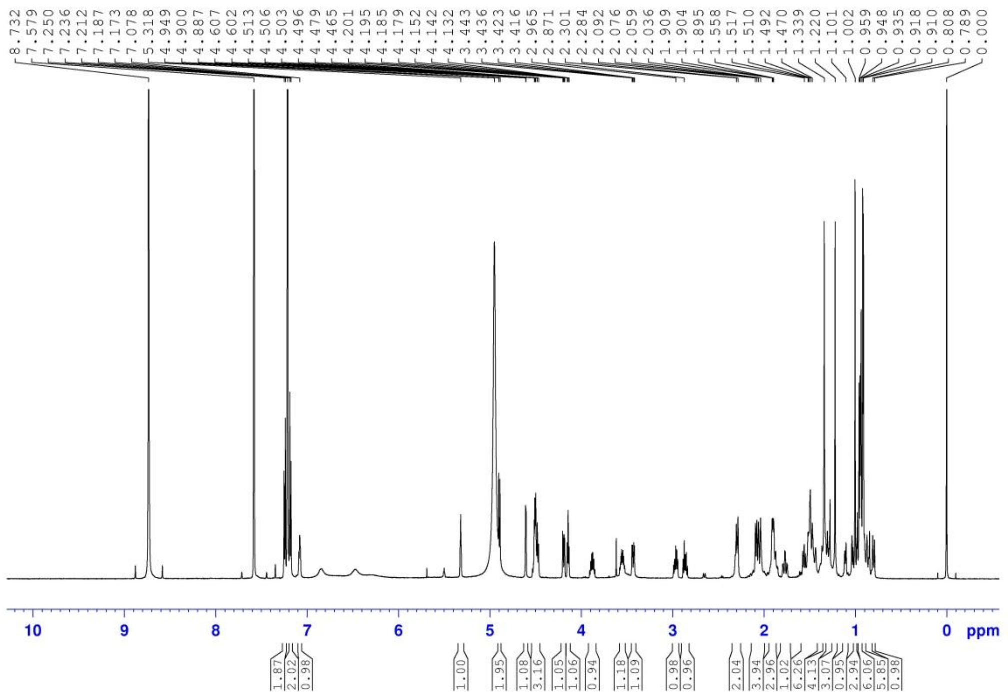

^{1}H NMR of compound **5c**

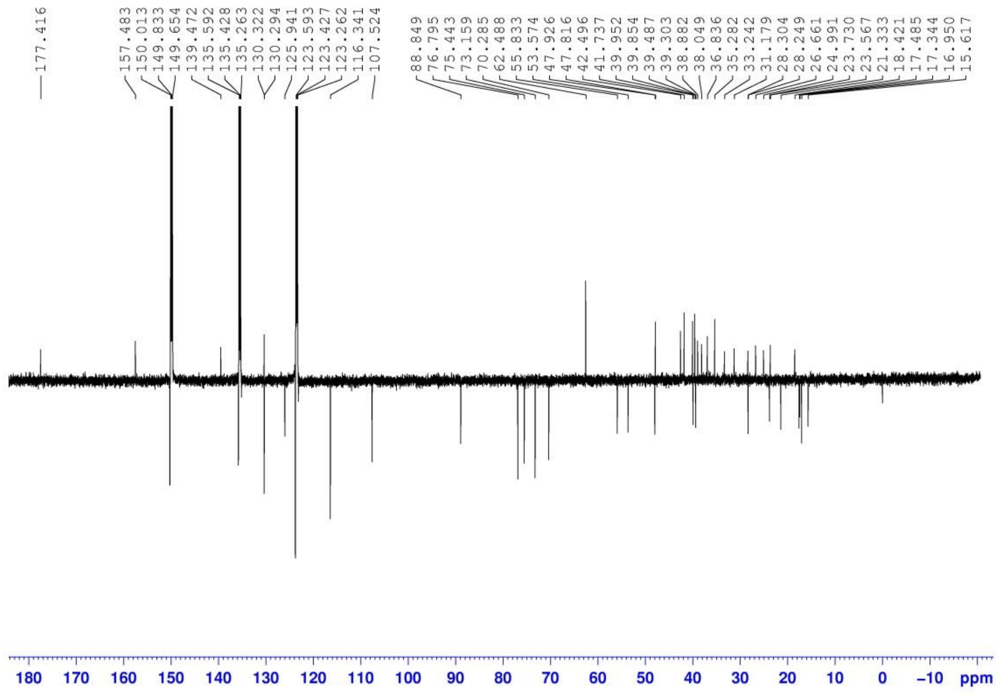

^{13}C NMR of compound **5c**

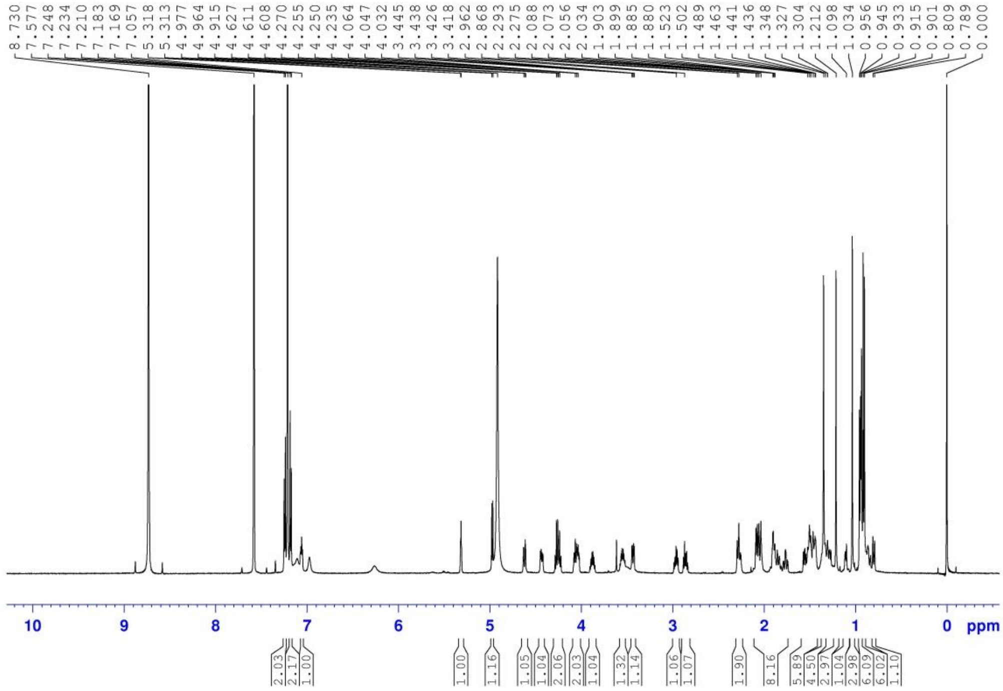

^{1}H NMR of compound **5d**

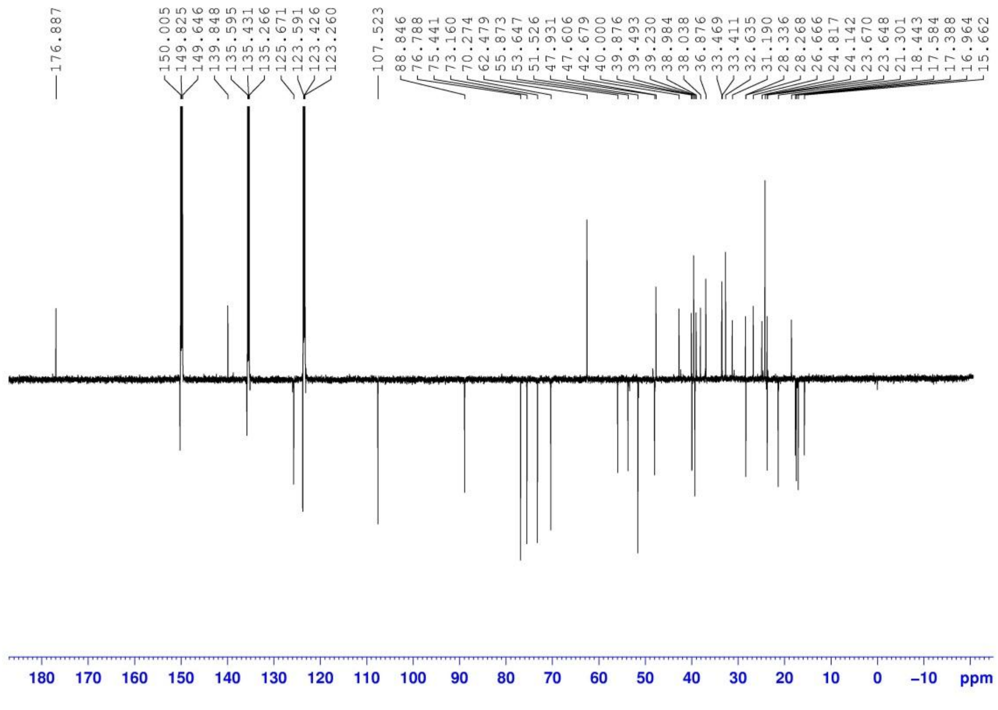

^{13}C NMR of compound **5d**
